# Metal sensing-carbon dots loaded TiO_2_-nanocomposite for photocatalytic bacterial deactivation and application in aquaculture

**DOI:** 10.1038/s41598-020-69888-x

**Published:** 2020-07-30

**Authors:** Rajaiah Alexpandi, Chandu V. V. Muralee Gopi, Ravindran Durgadevi, Hee-Je Kim, Shunmugiah Karutha Pandian, Arumugam Veera Ravi

**Affiliations:** 1 Lab in Microbiology and Marine Biotechnology, Department of Biotechnology, School of Biological Sciences, Alagappa University, Karaikudi, 630 003 India; 2 Lab in Laser and Sensor Application, School of Electrical and Computer Engineering, Pusan National University, Busandaehak-ro 63 beon-gil, Geumjeong-gu, Busan, 46241 South Korea

**Keywords:** Microbiology, Nanobiotechnology

## Abstract

Nowadays, bioactive nanomaterials have been attracted the researcher’s enthusiasm in various fields. Herein, *Diplocyclos palmatus* leaf extract-derived green-fluorescence carbon dots (DP-CDs) were prepared using the hydrothermal method. Due to the strong fluorescence stability, the prepared DP-CDs were coated on filter-paper to make a fluorometric sensor-strip for Fe^3+^ detection. After, a bandgap-narrowed DP-CDs/TiO_2_ nanocomposite (DCTN) was prepared using the methanolic extract of *D. palmatus.* The prepared DCTN exhibited improved photocatalytic bacterial deactivation under sunlight irradiation. The DCTN-photocatalysis slaughtered *V. harveyi* cells by the production of reactive oxygen species, which prompting oxidative stress, damaging the cell membrane and cellular constituents. These results suggest the plausible mode of bactericidal action of DCTN-photocatalysis under sunlight. Further, the DCTN has shown potent anti-biofilm activity against *V. harveyi*, and thereby, DCTN extended the survival of *V. harveyi*-infected shrimps during the in vivo trial with *Litopenaeus vannamei*. Notably, this is the first report for the disinfection of *V. harveyi*-mediated acute-hepatopancreatic necrosis disease (AHPND) using nanocomposite. The reduced internal-colonization of *V. harveyi* on the hepatopancreas as well as the rescue action of the pathognomonic effect in the experimental animals demonstrated the anti-infection potential of DCTN against *V. harveyi*-mediated AHPND in aquaculture.

## Introduction

The tainting of water bodies by heavy metals has become a severe ecological threat globally^1^. The development of exceedingly selective sensors has been attracting vast welfare for scrutinizing heavy metals due to their substantial environmental impacts because of severe toxicity and mobility^2^. Among various heavy metals, Iron (Fe^3+^) is the third most abundant element on the earth and considered as a crucial transition metal in cellular systems, which plays an important role in biological systems^3^. Furthermore, both deficiency and overabundance of Fe^3+^ are associated with serious diseases in humans^4^. Normally, this metal can be detected through various analytical techniques, including atomic absorption spectroscopy (AAS), mass spectrometry (MS), emission spectroscopy (ES), gas chromatography (GC), and voltammetry. However, these techniques are expensive, time-consuming, and inaccessible worldwide, particularly in developing countries^5^. Therefore, some fluorescent sensors, such as organic dyes, quantum dots (QDs), metal nanoclusters, and metal–organic framework probes have been reported for metal sensing^6^. Among these sensors, QDs have great attention due to their high aqueous solubility, robust chemical inertness, easy functionalization, high resistance to photo-bleaching, low toxicity, and good biocompatibility^7^. In particular, carbon quantum dots (CQDs) are innovative semiconductor carbon nanoparticles with a size of less than 10 nm that are mostly self-processed with sp^2^ carbon, oxygen, and other doped heteroatoms^8^. CQDs have pulled in expanding consideration in various fields because of their exceptionally stable fluorescence, photo-stability, quantum yield, low cost, water solubility, and positive biocompatibility^9^. Further, CQDs are highly established fluorescent-materials as promising candidates for a wide scope of potential applications, including metal sensors, bio-imaging, photocatalysts, and optoelectronic devices^10^. Due to the environmental compatibility, the CQDs emerge to be the best alternative to Cd^2+^/Pb^2+^-based QDs in electroluminescent LEDs and also considered as next-generation luminophore for future LEDs^11^. In addition, CQDs are exclusively utilized for glucose sensing, DNA testing, and dopamine sensing through electrochemical approach^12^. In recent, CQDs are used as efficient fluorophores for high-performance tandem-luminescent solar concentrators^13–15^.

A variety of methods has been utilized to synthesize CQDs, including hydrothermal, electrochemical, microwave, discharge-emission, plasma-treatment, and laser-ablation mediated synthesis^16^. Among these, hydrothermal method offers several advantages such as relatively one-step synthetic procedure, inexpensive, environmental friendliness, and high dispersion in solution^4^. In the present study, a biocompatible CQDs were synthesized from the aqueous leaf extract of *Diplocyclos palmatus* through hydrothermal approach. *D. palmatus* is a creeper-type tropical medicinal plant, mainly distributed in Malaysia, Australia, Africa, South China, and India. In previous, our group has been reported their protective efficacy on *Serratia marcescens* infection as well as anti-photoaging property using *Caenorhabditis elegans* model^17^.

On the other hand, the bacterial contamination in water bodies is one of the critical human health issues around the world^18^. Similarly, the bacterial contamination in aquaculture has practiced relatively many bacterial diseases owing to the lack of suitable treatment, resulting in cause serious economic loss and affecting sustainability of aquaculture industries^19^. Importantly, Vibrio species such as *Vibrio harveyi*, *V. vulnificus*, *V. parahaemolyticus*, and *V. alginolyticus* are frequently present in the seawater, and thereby associated with a number of infectious diseases, which are affecting a wide range of aquatic creatures including fishes, crustaceans, and molluscs^20^. Notably, *V. harveyi* is a gram-negative serious pathogen can able to cause several diseases to fish and shrimp, particularly *Penaeus monodon* and *Litopenaeus vannamei*^21^. This bacterium has well renowned to cause diseases such as acute hepatopancreatic necrosis disease (AHPND), vibriosis, septic hepatopancreatic necrosis (SHPN), skin ulcer, gastroenteritis, vasculitis, Bolitas negricans, eye lesions, and deep dermal lesions^22^. The biofilm formation and virulence factor production by bacterial pathogens attributed to their pathogenesis, which regulated by cell-density dependent gene expression, known as quorum sensing^23^. Therefore, antibiotics have been used to control bacterial infections in aquaculture so far. However, as the development of drug-resistance in bacterial pathogens due to the frequent use of antibiotics, a promising alternative approach is needed to control bacterial diseases in aquaculture^24^.

TiO_2_-based photocatalysis has offered an innovative platform to annihilate bacterial contamination in the water bodies using light irradiation^25^. However, the practical applications of TiO_2_ in solar-light is confined because of the wide bandgap (~ 3.2 eV), which requires UV-light (λ ≤ 387 nm) for its activation^26^. Therefore, modulating the bandgap absorption to the visible region is needed for practical functioning under direct sunlight^26^. Since UV-light comprises only 4–5% of the solar spectrum, whereas 40% of solar-light is in the visible region^27^. Hence, in the present study, a bandgap-narrowed carbon dots/TiO_2_ nanocomposite (DCTN) was prepared using the *D. palmatus* methanolic extract for photocatalytic bactericidal applications. Due to the antibiofilm behaviour against *V. harveyi*, the prepared nanocomposite also subjected to evaluating for its anti-infection potential against acute hepatopancreatic necrosis disease (AHPND) in *Litopenaeus vannamei* for valuable application in aquaculture.

## Results and discussion

### Structural and optical properties of synthesized DP-CDs

**Figure 1:**
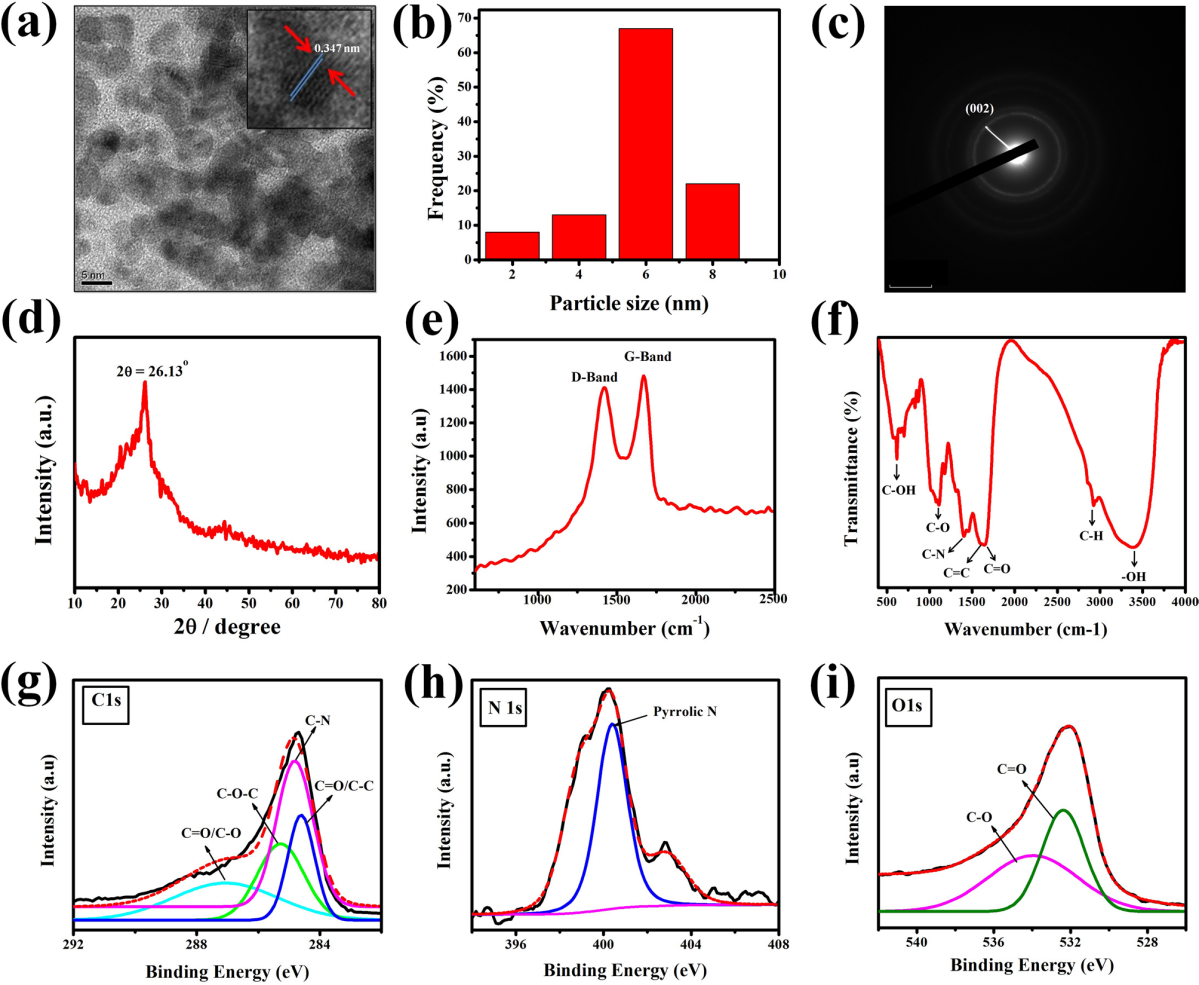
(**a**) HR-TEM images of the structural characterization of the as-prepared DP-CDs (Inset: Lattice d-spacing of 0.347 nm represents the (002) diffraction plane of sp^2^ carbon. (**b**) DLS measurement of DP-CDs in aqueous solution. (**c**) SAED pattern of individual particles of the DP-CDs. (**d**) XRD pattern of the DP-CDs revealed a (002) peak at 26.13° 2θ with a d-spacing of 3.49 Å, confirming the graphitic nature of the prepared DP-CDs. (**e**) Raman spectroscopic investigation of the defects or disorder in DP-CDs. (**f**) FT-IR analysis of functional groups on the surface of the DP-CDs. (**g**) C1s, (**h**) N1s and (**i**) O1s XPS high-resolution spectrum of DP-CDs.

In Fig. 1a, the HR-TEM analysis revealed the structural morphology of the prepared DP-CDs, wherein DP-CDs were spherical with a uniform size. Then, DLS analysis showed that the DP-CDs were mostly eight nanometers size (Fig. 1b). In Fig. 1c, the SAED pattern of an individual particle indicated the crystallinity of the DP-CDs with a lattice parameter of 0.347 nm, corresponding to the (002) diffraction plane of graphitic (sp^2^) carbon^28^. XRD analysis revealed a broad (002) peak at 26.13° 2θ with a d-spacing of 3.49 Å, which confirmed the graphitic nature of the DP-CDs (Fig. 1d). In addition, the Raman spectrum characterized the defects or disorders in DP-CDs (Fig. 1e). The relative intensity of the D-band (amorphous) and G-band (crystalline) was I_D_/I_G_ = 0.842, which showed that the synthesized DP-CDs were partially amorphous because of the presence of hydroxyl functional groups. The FT-IR spectrum showed the functional groups present on the surface of DP-CDs in Fig. 1f. The characteristic absorbance peaks at 3389 cm^–1^, 2925 cm^–1^, 1718 cm^–1^, 1619 cm^–1^, 1405 cm^–1^, 1181 cm^–1^, and 618 cm^–1^ were detected on the surface of the DP-CDs, indicating the presence of –OH stretching, C–H stretching, C=O stretching, C=C stretching, C–N stretching, C–O stretching, and C–OH vibrations, respectively^29^. In addition, XPS was used to examine the constituents of the surface functional groups of DP-CDs. In Fig. 1g, the high-resolution C1s XPS spectrum of DP-CDs was deconvoluted into four peaks at 284.7 eV, 285.1 eV, 285.6 eV, and 287.1 eV, which were assigned to C=C/C–C, C–N, C–O–C, and C=O/C–O bonds, respectively. The N1s spectrum displayed a peak at 400.7 eV, which denotes the pyrrolic N atoms (Fig. 1h). The O1s spectrum revealed two peaks at 532.4 eV and 535.1 eV, which indicates the presence of C=O and C–O (Fig. 1i). Moreover, the obtained FT-IR and XPS data reveal the surface wettability of the prepared DP-CDs. The presence of hydroxyl and carboxyl (hydrophilic) groups in the surface of DP-CDs, which makes surfaces super-hydrophilic nature^30–32^. Hence, the hydrophilic surface-wettability nature of DP-CDs is the reason for superior water solubility in aqueous solution^33^. Further, it is known that when plant extracts are hydrolyzed at high temperature, intermolecular and/or intramolecular dehydration will occur between the –OH, –COOH, and –H groups present in leaf extract and further polymerization, carbonization, and aromatization processes will lead to the formation of aromatic sp^2^ carbon^34^.

**Figure 2:**
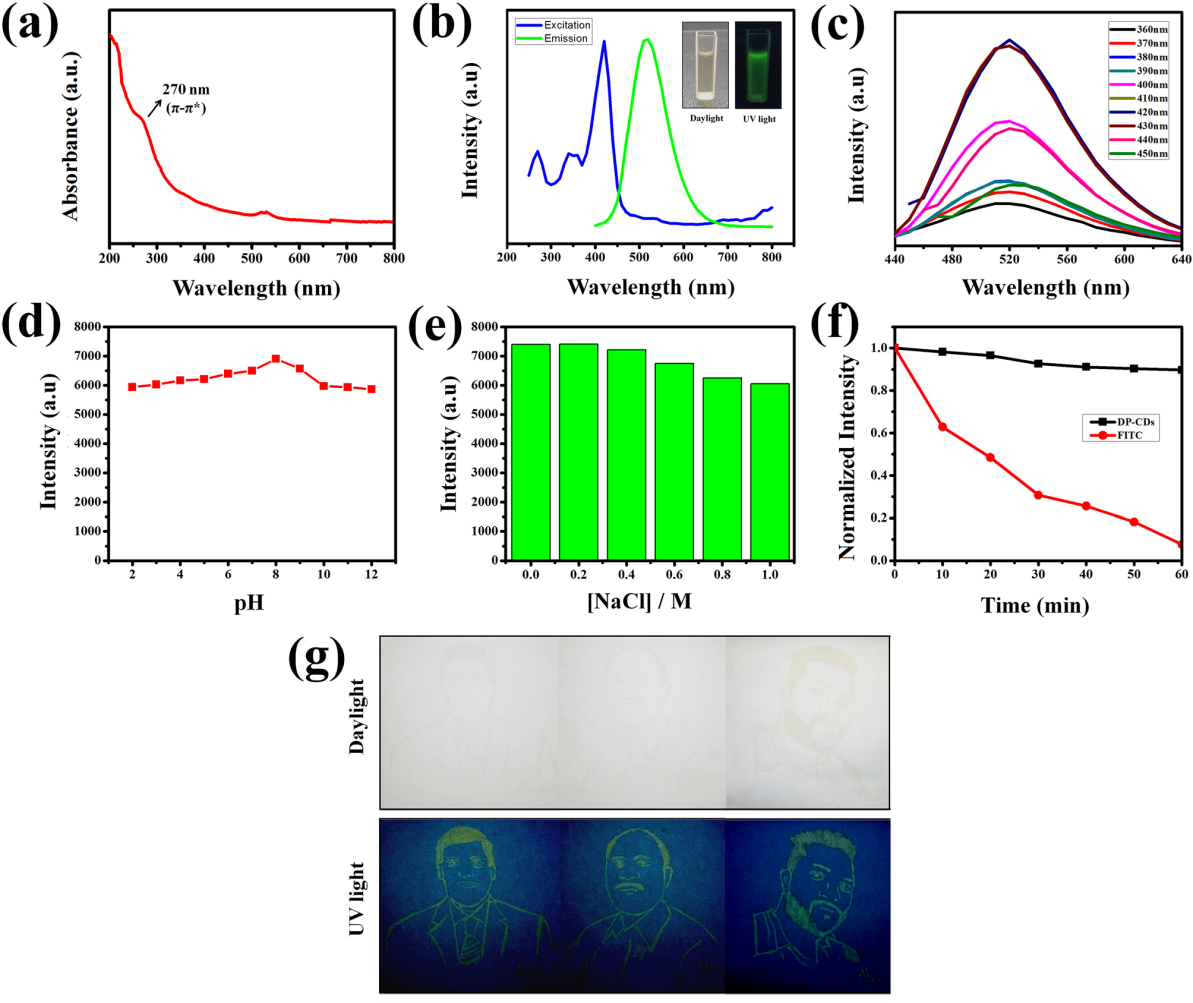
(**a**) UV–Vis absorbance spectrum of DP-CDs. (**b**) Fluorescence (FL) spectrum of DP-CDs (Inset shows a visual photograph of FL emission under daylight and UV-light irradiation). (**c**) FL emission spectra of DP-CDs at different excitation wavelengths with a 10 nm increments, from 360 to 460 nm. (**d**) FL intensity of DP-CDs under different pH conditions. (**e**) FL intensity of DP-CDs under various salt concentrations from 0 to 1 M in solution. (**f**) FL stability of DP-CDs under photoleaching with UV-irradiation. (**g**) Photographs showing FL emission of DP-CD-painted drawing of the authors (SKP, AVR, and RA) on filter paper.

In Fig. 2a, the UV–Vis absorption spectrum revealed a peak centered at approximately 270 nm, which was assigned to the π–π* transitions of aromatic C=C bonds in sp^2^ hybridization, and is a fingerprint of CQDs structures^28^. The fluorescence (FL) intensity of the DP-CDs under different excitation wavelengths, ranging from 360 to 450 nm, was measured. The obtained spectra demonstrated the maximum emission of 520 nm at 420 nm, which reveals the excitation-dependent emission of DP-CDs (Fig. 2b, c). Furthermore, the quantum yield of the DP-CDs was 31.4%. In addition, the stability of the DP-CDs under various pH conditions, high ionic strengths, different temperatures and various solutions was examined. The obtained result shows that the DP-CDs exhibited invariable FL intensity under the pH range of 2–12 (Fig. 2d). Similarly, DP-CDs maintained their intensity while increasing the ionic strength (Fig. 2e). In addition, the FL stability of DP-CDs under different temperatures as well as various solvents was analyzed. As shown in Fig. S1, the DP-CDs exhibits reduced-FL intensity while increasing temperature, which indicated the temperature-dependent FL emission of the DP-CDs. Moreover, the DP-CDs have stable FL emission under various solutions such as water, methanol, ethanol, acetone, chloroform, and petroleum ether (Fig. S2). It shows that the high FL emission was observed in water as well as methanol solution compared to other solutions. On the other hand, the DP-CDs showed a constant intensity even up to 1 h of UV-irradiation, whereas the well-known FL probe, fluorescein isothiocyanate (FITC) showed a decrease in intensity (Fig. 2f), which confirmed the photo-stability of the DP-CDs. In addition, the DP-CDs exhibited stable FL emission on filter paper and maintained their stability for a long time, as shown in Fig. 2g.

### On–off based Fe^3+^ sensing of DP-CDs coated fluorometric sensor-strip

**Figure 3:**
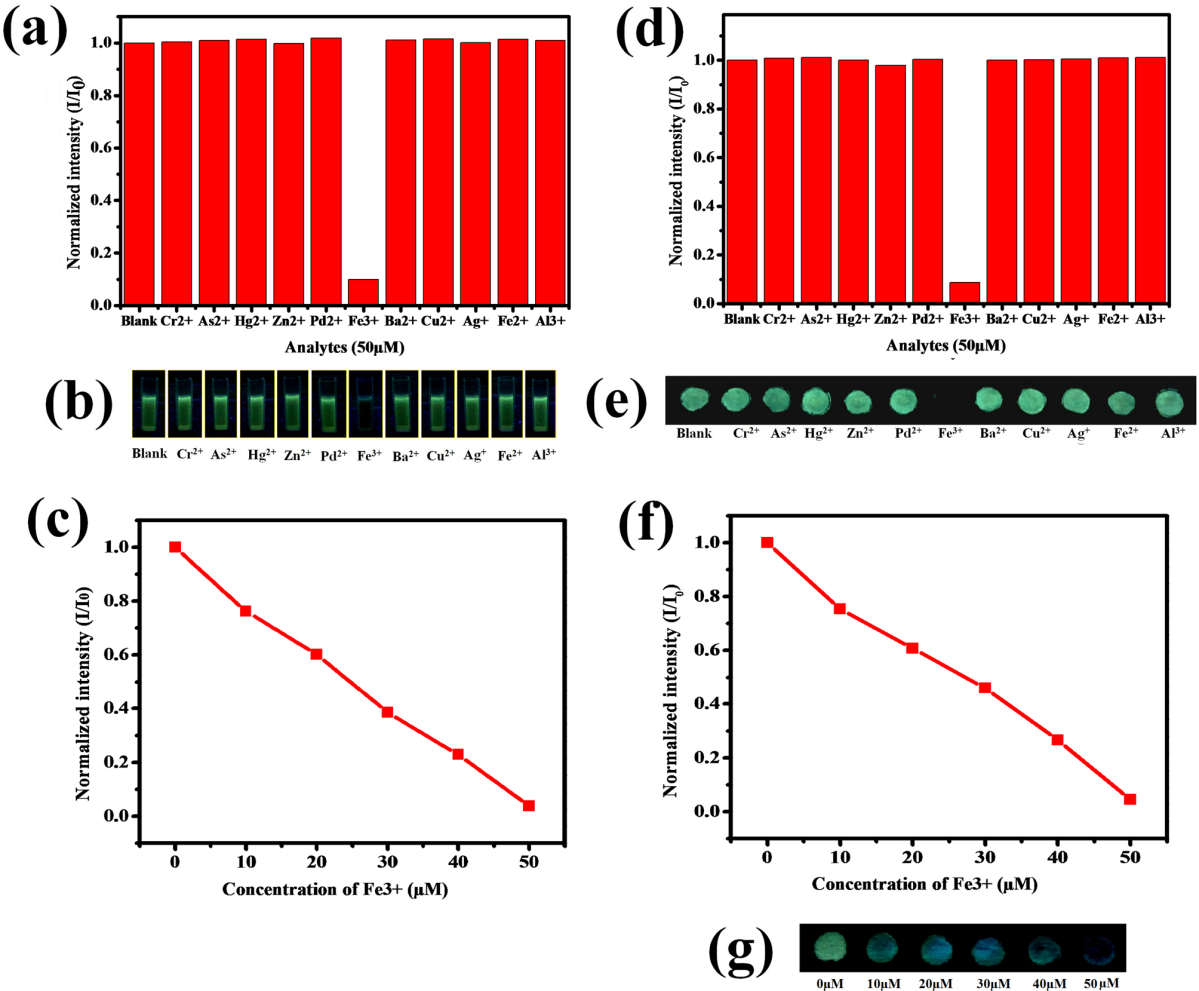
(**a**) Selectivity experiment on the changes in FL intensity (I/I_0_) of DP-CDs solution in the presence of different ion solutions. (**b**) Optical images of the DP-CD solution in the presence of these metal ions (50 µM). (**c**) FL response of DP-CDs solution in the presence of different concentrations of Cd^2+^ and Fe^3+^ (I/I_0_ corresponds to the changes in FL intensity in the absence and presence of Cd^2+^ and Fe^3+^ ions in solution). (**d**) Selectivity experiment of the DP-CDs in filter paper-based sensor strip. The graph shows the normalized intensity of DP-CDs after dropping of various ions solutions (50 µM) on the reaction zone. (**e**) Optical image of the FL intensity of DP-CDs sensor strip in the presence of different metal ions under UV-light captured by a Canon DSLR camera. (**f**) The graph represents the FL intensity changes (I/I_0_) of DP-CDs in the presence of different concentrations of Cd^2+^ and Fe^3+^ on the prepared DP-CD-coated sensor strips. (**g**) Optical images showing the sensitivity experiment for the identification of a low detection limit of DP-CDs sensor strip toward Cd^2+^ and Fe^3+^ ions.

Stable FL intensity of carbon dots could be useful to detect heavy metals in the form of fluorescence probe^28^. In order to predict the selectivity of the prepared DP-CDs in solution, the variation in FL intensity (I–I_0_) of DP-CDs was calculated in the presence of various metal ions (50 µM), including Cr^2+^, As^2+^, Hg^2+^, Zn^2+^, Pd^2+^, Fe^3+^, Ba^2+^, Cu^2+^, Ag^+^, Fe^2+^, and Al^3+^ (Fig. 3a, b). The result showed that Fe^3+^ abruptly quenched the FL intensity of DP-CDs and insignificant changes were observed with other ions. Then, the sensitivity of DP-CDs towards the concentration of Fe^3+^ was assessed. The sensing efficiency (changes in I/I_0_) of DP-CDs showed a good linear relationship with the Fe^3+^ concentrations (Fig. 3c), and the limit of detection for Fe^3+^ ions in solution was 10 μM. The FL emission of DP-CDs arises from radiative recombination under excitation^35^. Further, when binding with metal ions, the valence electrons in the metal ions that give sensitivity by forming strong interactions among the CQDs^28^. Herein, the high selectivity of Fe^3+^ ions probably due to the high affinity with the surface carboxyl groups (C–OH, C–O, and C=O) of the DP-CDs and thereby, forming a complex between Fe^3+^ ions and DP-CDs^36^. The complex formation by Fe^3+^ ions might affect the surface energy traps of the DP-CDs, which accelerates the non-radiative electron/hole recombination via an effective electron transfer process, and resulting cause FL quenching ultimately^28^, as shown in Fig. 8 Scheme-1.

Then, filter paper-based DP-CDs coated fluorometric sensor-strip was designed and made by drawing circular reaction pots using a wax liner^37^, as shown in Fig. 8 Scheme-2. Because, the paper-based analytical device (PAD) is a potential diagnostic tool due to its low-cost, user-friendliness, biodegradability, ease of fabrication, biocompatibility, and requirement of a smaller workforce without an external pump or power supply^38–40^. The selectivity of the prepared sensor-strip towards different metal ions was examined. Figure 3e shows digital photographs of the FL intensity of a reaction zone of a sensor-strip toward different ions. The FL intensity was captured using a Canon EOS 90D DSLR camera and quantified using Image-J software. As shown in Fig. 3d, e, similar FL quenching was observed with Fe^3+^ in sensor-strip, which was in agreement with our previous result. During the sensitivity trial, the intensities of the reaction zones in the sensor-strip decreased gradually in the presence of increasing concentrations of Fe^3+^ ions (Fig. 3f, g). The minimum detection limit was reported to be 10 µM based on visual differentiation and Image-J quantification. In conclusion, this part of the work suggests that the prepared fluorometric sensor-strip could act as an easy-handling and promising microfluidic-device for the onsite detection of Fe^3+^ ions.

### Synthesis and characterization of DP-CDs loaded TiO_2_ nanocomposite (DCTN)

**Figure 4:**
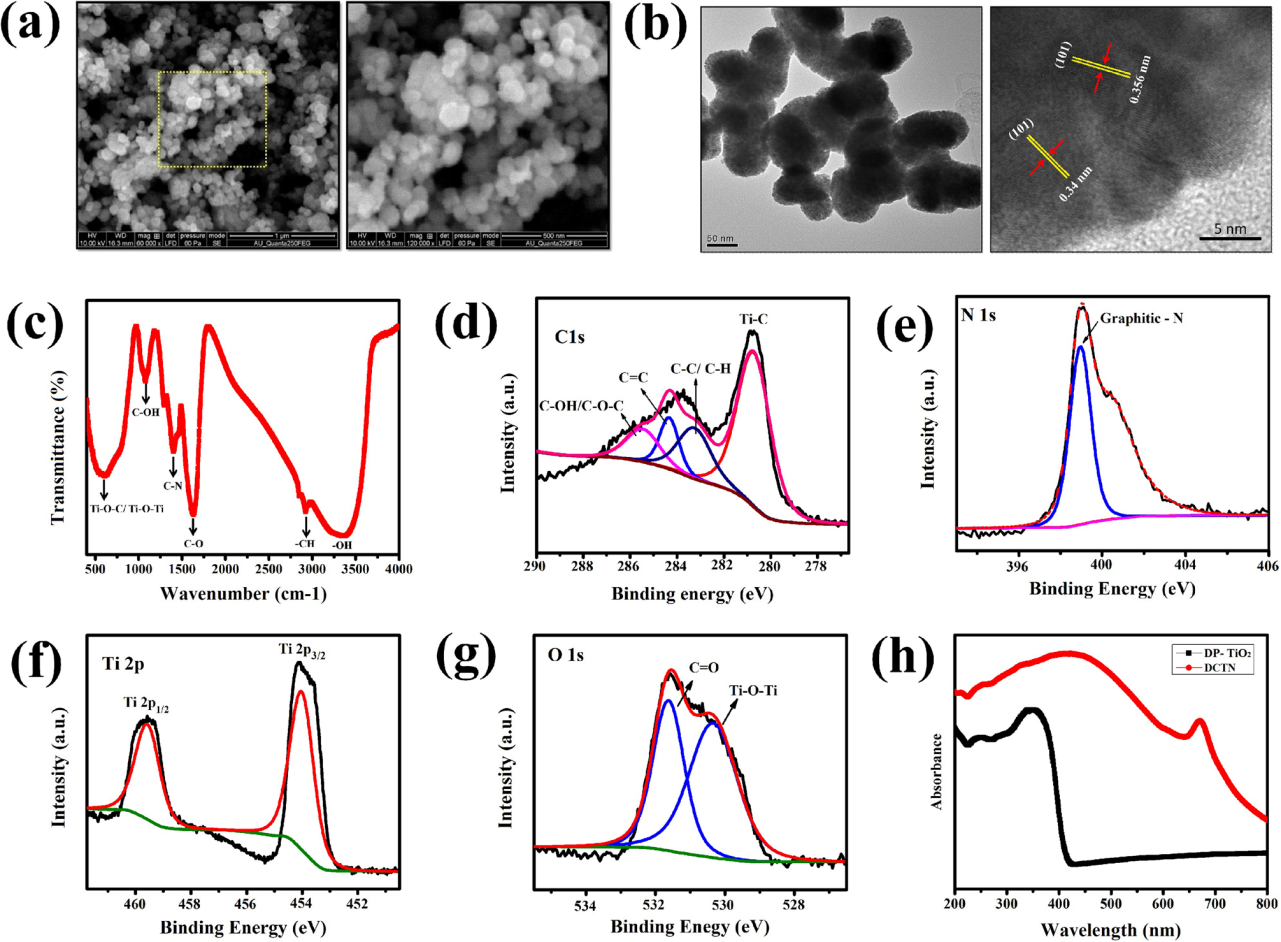
(**a**) FE-SEM and (**b**) HR-TEM analysis of the DCTN. (**c**) FT-IR analysis of functional groups on the surface of the DCTN. (**d**) C1s, (**e**) N1s, (**f**) Ti 2p and (**g**) O1s XPS high-resolution spectrum of DCTN. (**h**) UV-DRS absorbance spectra of DCTN and DP-TiO_2_.

On the other hand, a bandgap-narrowed DP-CDs/TiO_2_ nanocomposite (DCTN) was successfully prepared using *D. palmatus* methanolic leaf-extract as a capping and stabilizing agent under hydrothermal condition. Our previous finding has reported the phytochemical profiling of *D. palmatus* methanolic leaf-extract using gas chromatography-mass spectrometry (GC–MS) analysis^17^. Based on the report, the major compounds such as palmitic acid, tocopherols, and phytol are antioxidant compounds having –OH functional groups, which acted as reducing and capping agents during the nanocomposite synthesis. In characterization studies, the FE-SEM analysis revealed the spherical shape of the prepared nanocomposite in Fig. 4a. Further, the HR-TEM analysis clearly showed that the coupled structure of the DCTN was spherical (Fig. 4b). Then, the lattice spacing of the 0.347 nm of (002) plane and 0.356 nm of (101) plane established the presence of DP-CDs and TiO_2_ in the nano-spheres. In addition, AFM analysis also confirmed the spherical-structure of the prepared DCTN in the 3D arrangement and revealed the size, which was ~ 78.3 nm on average (Fig. S3). The XRD patterns revealed reflections at 27.4°, 36.1°, 39.1°, 41.2°, 44.1°, 54.4°, 56.6°, 63.1°, 64.1°, and 69.8° 2θ, which were indexed to the (110), (100), (101), (200), (111), (210), (211), (220), (002), (310), and (112) crystallographic planes of rutile TiO_2_ (Fig. S4). This data clearly showed that doping of DP-CDs does not alter the lattice structure of TiO_2_ during composite preparation. In the FT-IR spectrum, the characteristic peaks at 3389 cm^–1^, 2925 cm^–1^, 1618 cm^–1^, 1405 cm^–1^, and 1181 cm^–1^, indicate the presence of –OH, C–H, C–O, C–N, and C–OH groups, respectively (Fig. 4c). The broad absorption below 1000 cm^−1^ represents the combination of Ti–O–Ti and Ti–O–C vibrations^41^. This suggests that coupling between the TiO_2_ and DP-CDs developed through these bonds. The high-resolution C1s XPS spectrum of DCTN was deconvoluted into four peaks at 280.9 eV, 283.1 eV, 284.6 eV, and 285.8 eV, which were assigned to the Ti–O–C, C–C/C–H, C=C, and C–OH/C–O–C bonds, respectively (Fig. 4d). The N1s spectrum has a peak at 399.1 eV, which represents the C–N bonding (Fig. 4e). In the Ti 2p high-resolution spectrum, two peaks at 458.7 and 464.9 eV indicated the binding energies of Ti2p 3/2 and Ti2p 1/2, respectively (Fig. 4f). The O1s spectrum showed two peaks at 530.1 eV and 531.6 eV, which were assigned to Ti–O–Ti/Ti–O–C and C=O bonds, respectively (Fig. 4g). Overall, the XPS and FT-IR data suggest the strong interaction between DP-CDs and TiO_2_ via the formation of Ti–O–C bonds. The UV-DRS spectra show that the DP-TiO_2_ nanoparticles did not absorb in the visible region between 400 and 800 nm, whereas DCTN exhibited an extended absorbance in the visible light region (Fig. 4h), which indicates that DCTN has better photocatalytic activity under direct sunlight^42^. In addition, the bandgap of DCTN was estimated to be 2.87 eV using the Tauc equation, which means that DCTN has a smaller bandgap than DP-TiO_2_ (3.25 eV) and P25 TiO_2_ (3.15 eV) (Fig. S5).

### Photocatalytic bacterial-deactivation of DCTN under sunlight

**Figure 5:**
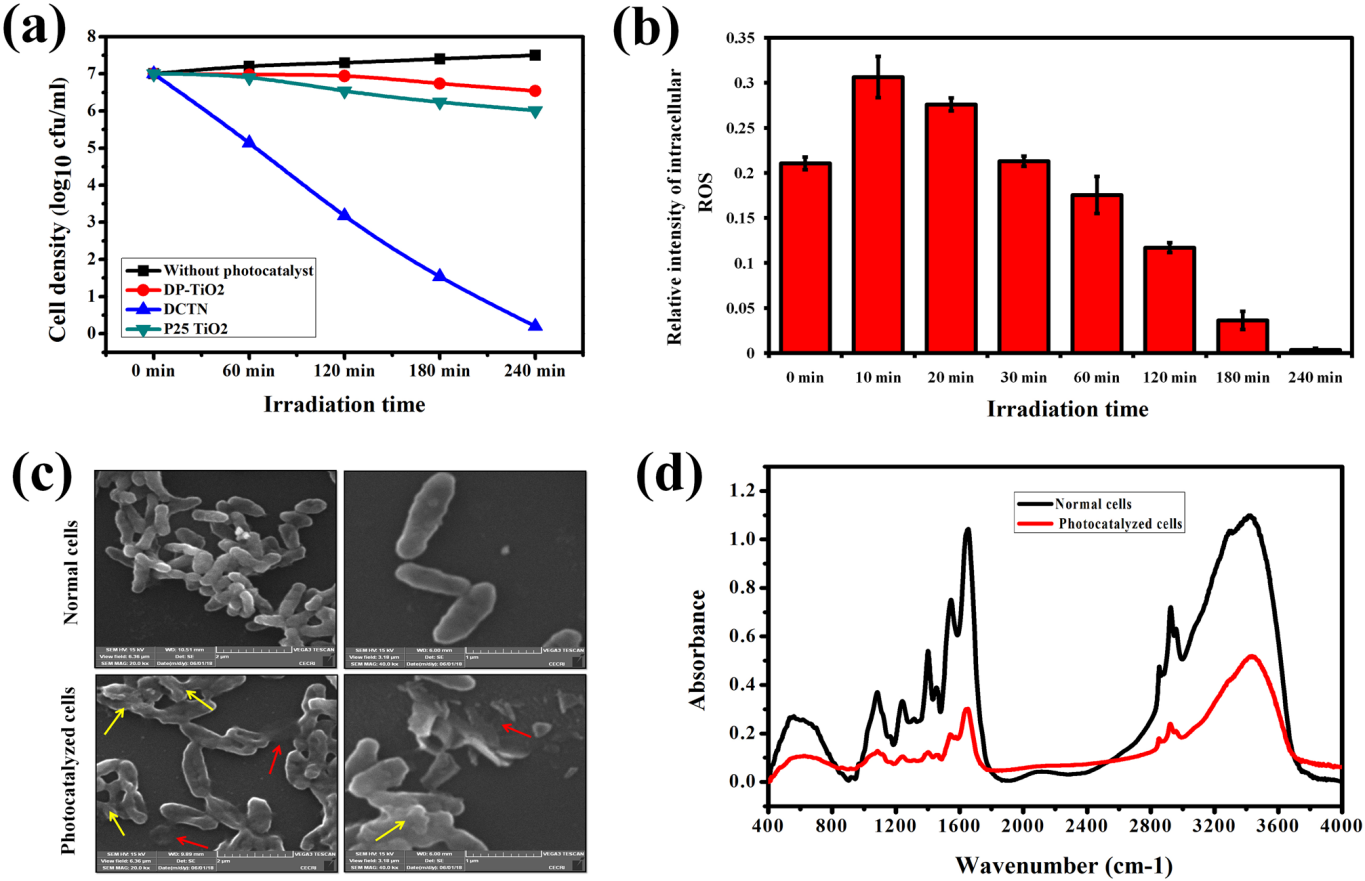
(**a**) Photocatalytic deactivation of *V. harveyi* over DCTN at different time points under sunlight exposure. (**b**) The graph represents the intracellular ROS level in *V. harveyi* during DCTN photocatalysis under sunlight (detected by DCFDA method). (**c**) FE-SEM analysis of DCTN photocatalysis-induced cell damage in *V. harveyi* upon sunlight irradiation (240 min). The yellow color arrow indicates the membrane damage and the red color arrows indicate the leakage of cellular components. (**d**) FT-IR analysis of the disruption of cellular components upon DCTN photocatalysis in *V. harveyi* upon sunlight irradiation. The result of the FT-IR spectra illustrates the reduction in the regions, such as (**a**) glycoside linkages of polysaccharide molecules in the cell membrane (600–800 cm^−1^), (**b**) bacterial membrane phospholipids (1087 and 1238 cm^−1^), (**c**) amide linkage from proteins and peptides (1550–1645 cm^−1^), and (**d**) fatty acids in the cell membrane (2700–3100 cm^−1^) in the photocatalyzed *V. harveyi*.

The photocatalytic bacterial deactivation of DCTN under sunlight was evaluated using *Vibrio harveyi* (MTCC 7771) as a model bacterium. It was found that the time-dependent bacterial killing was observed in the presence of DCTN photocatalyst under sunlight irradiation (Fig. S6). In Fig. 5a, the result depicted that the untreated *V. harveyi* barely shows self-degradation (~ 3%), whereas the complete killing of *V. harveyi* was noted within 240 min in the presence of DCTN photocatalyst. The bacterial degradation efficacy of DCTN under sunlight was higher than that of commercial P25-TiO_2_ and prepared DP-TiO_2_. Moreover, there was no bacterial killing in the presence of DCTN, P25-TiO_2_, and DP-TiO_2_ under dark conditions. This result clearly revealed that the bacterial killing of *V. harveyi* accomplished by the photocatalytic activity and not by their antibacterial activity of DCTN. In addition, the photocatalytic bactericidal activity of environmental water samples also performed. The obtained result shows that DCTN-photocatalysis significantly inactivated the bacterial load in the natural seawater (Fig. S7) as well as tap-water (Fig. S8), within 240 min under sunlight irradiation. complete.

Similarly, Bonnefond et al.^43^ have achieved bacterial deactivation of *Escherichia coli* within 240 min using acrylic/TiO_2_ nanocomposite under sunlight irradiation. Sharma et al.^44^ reported that TiO_2_–Fe_2_O_3_ nanocomposite has exhibited complete bacterial killing within 120 min under sunlight. In another hand, Lin et al.^45^ have deactivated *E. coli* cells within 360 min using iodine-modified TiO_2_ under visible light irradiation. Yadav et al.^46^ have successfully inactivated *E. coli* and *Salmonella abony* cells under 300 and 360 min of visible light irradiation, respectively. Ouyang et al.^47^ have effectively disinfected *E. coli* cells within 120 min using C_70_–TiO_2_ hybrid thinfilm under visible light irradiation. Similar to our finding, Zeng et al.^48^ have reported that carbon dots-TiO_2_-rGO nanocomposite rapidly destroyed *E. coli* cells within 60 min under simulated solar-light irradiation. Taking everything into account, the present study is the first study was reported the photocatalytic deactivation of aquatic pathogen-*V. harveyi* with its mechanism insights, which was achieved within 240 min under sunlight irradiation. Hence, due to the successful and efficient bacterial killing efficacy, the present study profoundly suggests the prepared DCTN as an proficient photocatalyst for water disinfection process using sunlight irradiation.

### Mode of ROS induced bactericidal action during DCTN photocatalysis

The active mechanisms of photocatalytic mediated bacterial deactivation using TiO_2_-based photocatalysis are well known. Briefly, the photogenerated active free radicals induce oxidative stress that damage the cell membranes as well as the cellular components, including proteins, lipids, enzymes, DNA, and other biomolecules^49^, and ultimately cause bacterial death, as shown in Fig. 8 Scheme-3. Therefore, the generation of ROS stress inside *V. harveyi* cells plays a pivotal role in the photocatalysis-mediated bacterial deactivation process^50^. Therefore, the intracellular ROS level in *V. harveyi* during DCTN-photocatalysis under sunlight irradiation was measured using a DCFDA-probe method. The result shows that the intracellular ROS level in the photocatalyzed cells increased extensively compared to the normal cells at the initial time point (Fig. 5b). Subsequently, the intensity of ROS decreased gradually with increasing experimental time, which indirectly confirming the bacterial death during DCTN-photocatalysis^51^. The predominant stimulation of intracellular ROS stress in *V. harveyi* cells indicates the ROS-mediated bacterial killing upon DCTN-photocatalysis under sunlight irradiation.

At the same time, the cell morphology was observed by SEM to better understanding the deleterious effect of ROS-induced bactericidal action on *V. harveyi*^52^. The SEM results revealed the uninterrupted, damage-free, and usual morphology of gamma-like bacteria in the control samples (Fig. 5c), whereas, broken cells along with remarkable changes, such as membrane damage caused by forming pits and holes in their cell membrane in the photocatalyzed samples, which are evidently visualized the cell-membrane dameges during the DCTN-photocatalysis. Further, the disruption of the cellular components was confirmed by FT-IR spectroscopy (Fig. 5d), which showed a significant decrease in the region of 600–800 cm^−1^, indicating the breakdown of glycoside linkages of polysaccharide molecules in the cell membrane^53^. The reduction at 1087 and 1238 cm^−1^ related to the breakdown of bacterial membrane phospholipids in photocatalyzed *V. harveyi*. The reduced intensity at 1550 and 1645 cm^−1^ denotes the damage to the amide-I and amide-II bonding of proteins and peptides^54^. The perceptible decrease in the intensity around 2750–3050 cm^−1^ indicates the damage to the fatty acid contents in the cell membrane in the photocatalyzed cells^17^. Overall, these results hypothesized that the inactivation of *V. harveyi* was accomplished mainly by the apparent damage of the cell membrane as well as the subsequent removal of cellular components by oxidative stress during the DCTN-photocatalysis.

### Identification of ROS generation during DCTN photocatalysis under sunlight

The prepared DCTN showed improved visible light absorption due to the narrowed bandgap compared to the commercial P25-TiO_2_ and prepared DP-TiO_2_, as shown in Fig. S5. The results of FT-IR and XPS analysis also revealed that the significant decrease in bandgap could be associated with the chemical bonding between TiO_2_ and DP-CDs by Ti–O–C bond formation in the DCTN. Several studies also reported that the presence of Ti–O–C bonding offers charge transfer and can extend light absorption to longer wavelengths^55,56^. At the same time, DP-CDs can act as an electron acceptor to trap the electrons from the conduction band of TiO_2_ and hinder electron–hole recombination, which leads to more charge carriers to produce reactive oxygen species (e.g., OH· and O_2_·−)^57^. These reactive species are very strong oxidants that promote bacterial deactivation under sunlight irradiation.

The formation of photocatalytically generated ROS by the DCTN photocatalyst under sunlight irradiation was confirmed using the 2,3-Bis(2-methoxy-4-nitro-5-sulfophenyl)-2H-tetrazolium-5-carboxanilide (XTT) assay and terephthalic acid (TA) method. In the XTT assay, O_2_·− reduces XTT to XTT-formazan and shows a specific absorption peak at 470 nm. The XTT-formazan concentration indicates the formation of reactive species (O_2_·−). The results showed that the formation of O_2_·− radicals in DCTN photocatalysis is much higher than that in DP-TiO_2_ and P25 TiO_2_ under sunlight irradiation (Fig. S9). Similarly, TA reacts with OH· radicals to form 2-hydroxyterephthalic acid (TA), which emits an FL signal at 420 nm^58^. The results showed that DCTN exhibited an enhanced FL intensity of TA compared to DP-TiO_2_ and P25 TiO_2_, which validates the enhanced production of OH· radicals under sunlight irradiation (Fig. S10).

### Anti-infection activity of DCTN against *V. harveyi-*caused acute hepatopancreatic necrosis disease (AHPND)

It is well-known that aquaculture is an important food-producing sector around the globe^59^. About 75% of the global production of aquatic-products comes from the Asian countries, including China, Thailand, Vietnam, Indonesia, and India^60^. However, the bacterial disease called acute hepatopancreatic necrosis disease (AHPND) mainly caused by *V. harveyi* intensely affects shrimp production, which leading to global economic losses^61^. During the AHPND pathogenesis, *V. harveyi* colonizes on the shrimp hepatopancreas (HP), in which it causing tissue destruction and dysfunction of the HP and digestive organs^62^. Hence, there is an increasing attention in the development of effective anti-infection agents to control the Vibrio mediated bacterial infections in aquaculture through anti-biofilm or anti-quorum sensing (anti-QS) approach in recent years^23,24^.

### In vitro anti-biofilm and anti-virulence activity of DCTN

**Figure 6:**
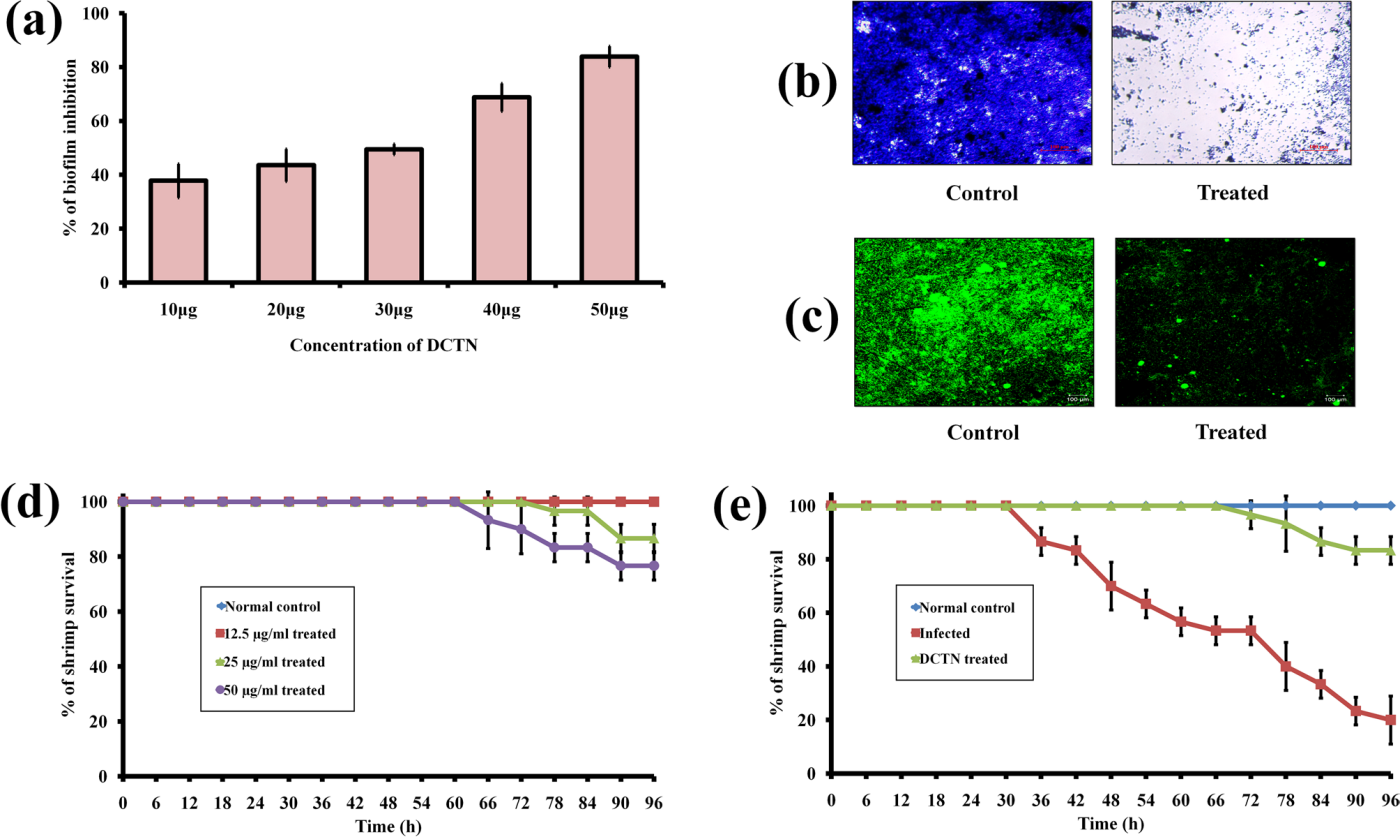
(**a**) Par graph represents the percentage of biofilm inhibition by DCTN on *V. harveyi*. (**b**) The image indicates the light microscopic observation of biofilm inhibition upon DCTN treatment. (**c**) CLSM analysis further endorses the anti-biofilm potential of DCTN against *V. harveyi* at 50 µg/ml concentration. (**d**) The graph reveals the survival percentage of shrimp (*P. vannamei*) in the presence of DCTN with various concentrations. (**e**) The graph shows the survival percentage of *V. harveyi* infected animals upon DCTN treatment at the selected dosage (12.5 µg/ml).

Biofilms are an aggregated growth pattern of bacterial pathogens, which acts vital role in their pathogenesis to cause infectious diseases to the host^63,64^. Hence, hindering biofilm formation by anti-biofilm agents has considered a promising mode to trim-down the infection rate in aquaculture^23^. Recently, Soowannayan et al.^65^ have successfully protected AHPND infected shrimps (*P. vannamei*) using Vibrio biofilm inhibitors. Therefore, the antibiofilm activity of the DCTN was assessed using in vitro assay^17^. The obtained result reveals that the DCTN effectively inhibited the biofilm formation of *V. harveyi* to the level of 85% at 50 μg/ml concentration (Fig. 6a). Further to confirm the anti-biofilm activity against *V. harveyi*, *in-situ* visualization of biofilms was performed using light microscopic and CLSM analysis^54^. In light microscopic results, the untreated *V. harveyi* has a thick coating of biofilms on glass slides; whereas, a perceptive diminution of biofilm cells was obtained in DCTN treated slides (Fig. 6b). Then, the dynamic complexity of biofilm architecture was observed using CLSM analysis^66^. The result depicts that the highly complex architecture was obtained in the untreated control sample; whereas, a reduced biofilm architecture was obtained in the DCTN treated sample (Fig. 6c), which strongly authenticated the anti-biofilm potential of DCTN against *V. harveyi*.

In addition, the bacterial pathogenicity also related to the secretion of QS-regulated extracellular virulence enzymes such as hemolysin and protease, which are facilitate the bacteria to defend against the host immune response^24^. In the current study, DCTN inhibited the hemolysin and protease production of *V. harveyi* to the level of 75 and 72%, respectively (Fig. S11). This section of the present study concluded that DCTN has the potential to inhibit QS-regulated biofilm formation as well as virulence factor production in *V. harveyi*.

### In vivo toxicity level of DCTN in *P. vannamei*

Before going to assess the in vivo anti-infection activity, the toxicity level of DCTN was studied using a survival assay with whiteleg shrimp (*Penaeus vannamei*)*.* It was found that DCTN has not shown any reduction in the survival of *P. vannamei* at 12.5 µg/ml concentration, whereas 80% of survival was observed at 50 µg/ml treatment (Fig. 6d). Hence, we have selected 12.5 µg/ml dosage for subsequent in vivo experiments**.** Further, the toxic level of commercial P25-TiO_2_ as well as DP-TiO_2_ at 12.5 µg/ml concentration was tested. The result shows that both DP-TiO_2_ and P25-TiO_2_ has significant toxicity to *P. vannamei* (Fig. S12)*.* So, this part of the work strongly suggests that the prepared DCTN is more biocompatible than commercial P25-TiO_2_ and DP-TiO_2_.

### In vivo anti-infection activity against *V. harveyi-*caused AHPND

**Figure 7:**
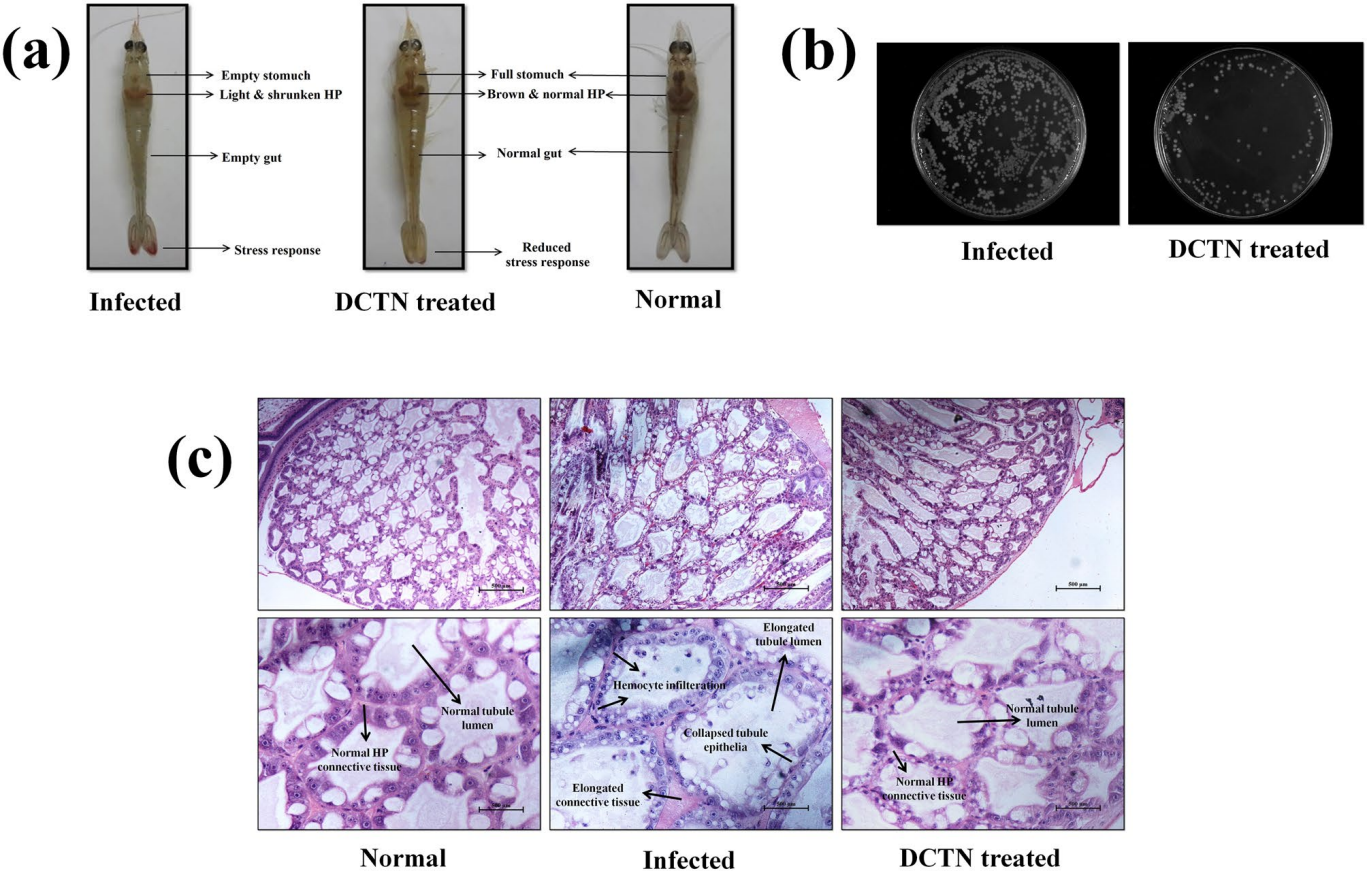
(**a**) Photographs reveal the pathognomonic symptoms of *V. harveyi*-caused AHPND in shrimp and rescue action of DCTN treatment. (**b**) The representative image for the reduction of *V. harveyi* colonization inside the HP by DCTN treatment. (**c**) The histopathology images of hematoxylin and eosin (H&E) stained hepatopancreatic (HP) tissues of the experimented shrimps.

To appraise the in vivo anti-infection efficacy of DCTN on *V. harveyi*-caused AHPND in *P. vannamei*, the survival assay was performed with infected animals. Figure 6e shows that the survival of infected shrimp without any treatment was 20%; whereas, the survival of shrimp treated with 12.5 µg/ml of DCTN was increased up to 83.33% in 96 h experiment. In Fig. 7a, the pathognomonic symptoms of AHPND such as pale and shrunken HP and empty gut were observed in the *V. harveyi* infected group^65^; wherein, these infectious lesions were significantly abridged in the DCTN treated group. This result evidently authorizes the in vivo disease protection efficacy of DCTN against *V. harveyi* caused AHPND.

The survival extension of infected shrimps consummated by hindering the *V. harveyi* accumulation inside the shrimp HP. Therefore, the *V. harveyi* accumulation inside the HP was analyzed using the CFU assay. The result displays a huge amount of *V. harveyi* colonization (8.63 × 10^4^ CFU) observed in the infected shrimp; whereas, significantly reduced bacterial colonization (1.89 × 10^4^ CFU) observed in the DCTN treated group (Fig. 7b). This result concluded that the DCTN-treatment effectively reduced the *V. harveyi* colonization, and thereby, rescued the infected shrimps from the infectious effect of AHPND.

In histopathological analysis, hematoxylin and eosin (H&E) stained HP tissues of shrimp reveal that the massive sloughed and elongated HP, collapsed tubule epithelia and severe hemocyte infiltration in the *V. harveyi* infected shrimp (Fig. 7c), which characterizes the factual pathognomonic symptoms of AHPND in shrimp culture^67^. In contrast, the HP tissue of treated with DCTN showed normal HP histology, which similar to the uninfected control group. Overall, the obtained results suggest that the bioactive DCTN can be exploited to care for acute hepatopancreatic necrosis disease in shrimp aquaculture.

## Conclusions

A simple, low-cost, and facile approach was developed to prepare fluorescent carbon dots using a plant extract with a quantum yield of 31.4%. The prepared DP-CDs showed strong FL stability and photo-stability. Consequently, the FL intensity decreased upon binding with Fe^3+^ions, which prompted the development of an uncomplicated on–off fluorometric sensor-strip for Fe^3+^ detection. Subsequently, DP-CDs/TiO_2_ hybrid-spheres (DCTN) were prepared for photocatalytic application. As expected, DCTN showed superior photocatalytic activity in bacterial deactivation under sunlight because of the narrowed bandgap. The DCTN effectively deactivated *V. harveyi* by the photogenerated ROS stress, which damaged the cell membrane and cellular components, resulting in bacterial death under sunlight irradiation. On the other hand, the DCTN exhibited admirable anti-biofilm activity against *V. harveyi.* Further, the DCTN not showed any significant toxicity in *P. vannamei,* which indicates that the prepared DCTN is more biocompatible than commercial P25-TiO_2_ and DP-TiO_2_. Consequently, the DCTN showed excellent disinfection activity against *V. harveyi*-induced AHPND in *P. vannamei*. The lifespan extension of *V. harveyi*-infected shrimps was achieved by reducing the internal accumulation of *V. harveyi* inside the HP upon the DCTN treatment. The histopathology study also validated the rescue action from the pathognomonic effects of *V. harveyi*-caused AHPND in the treated shrimps. Overall, the present study recommends that the prepared bioactive plant-derived carbon dots-TiO_2_ nanocomposite (DCTN) present a great prospect in photocatalytic water disinfection, along with an innovative biological-application in the treatment of *V. harveyi*-mediated acute hepatopancreatic necrosis disease for shrimp aquaculture.

## Methods

### Materials

The leaves of *D. palmatus* (L.) were shade dried and ground into a fine powder. Subsequently, 10 g of leaf powder was extracted sequentially using 100 ml of distilled water and methanol. The aqueous extract was used for carbon dot synthesis, and the methanolic extract was used as a capping and stabilizing agent for the nanocomposite synthesis. Titanium isopropoxide (C_12_H_28_O_4_Ti, > 97% purity) was purchased from Sigma-Aldrich (St. Louis, MO, USA) and used without further purification as a precursor for the carbon dot/TiO_2_ composite synthesis. All the chemicals and reagents used in the current study were purchased from Sigma-Aldrich. Whatman No.1 (#3) filter papers were used for the preparation of the sensor strip.

### Preparation and characterization of carbon dots

In the experiment, 100 ml of an aqueous extract of *D. palmatus* was transferred to a Teflon-lined autoclave and sealed tightly to maintain the intrinsic pressure. The autoclave was heated to 180 °C for 30 min. After a dark brown solution formed, it was allowed to cool to room temperature. Finally, the solution was centrifuged, and the supernatant was filtered through a 0.2 μm filter membrane to eliminate the micron-sized particles. The final solution was lyophilized to obtain solid carbon dots and stored at 4 °C for subsequent studies.

The structural morphology was investigated by high-resolution transmission electron microscopy (HRTEM, JEM 2011, JeolCo.). The particle size was observed using a Zeta-sizer (Nano ZS, Malvern Instruments, United Kingdom). The crystallinity was investigated by X-ray powder diffraction (XRD, PAN analytical X-Pert PRO). Raman spectroscopy was carried out using an imaging spectrograph STR 500 mm focal length laser Raman spectrometer at room temperature. The elemental composition was investigated by energy-dispersive X-ray (EDX, SU-70, Hitachi) attached to the FE-SEM (SU-70, Hitachi) instrument. The surface chemical composition was observed by X-ray photoelectron spectroscopy (XPS, PHI 5600ci) using AlKα radiation (hv = 1486.6 eV, Thermo Fisher Scientific). XPS curve fitting and elemental analyses were performed using a Casa XPS 2318 PR1-0 instrument. The surface functional groups were investigated using Fourier Transform Infrared (FT-IR, Thermo scientific iS4) spectroscopy using the KBr compressed pellet method. The fluorescence intensity of the DP-CDs was observed using a Shimadzu RF-5310 PC spectrofluorophotometer. The quantum yield (QY) of the DP-CDs was measured using quinine sulphate (QY: 54%; dissolved in 0.1 M H_2_SO_4_ aqueous solution) as a standard, and the yield was calculated using the following formula:

1 $$\varphi_{c} = \varphi_{s} \times \frac{{A_{s} }}{{I_{s} }} \times \frac{{I_{c} }}{{A_{c} }} \times \frac{{\eta_{c}^{2} }}{{\eta_{s}^{2} }}$$

where φ, A, *I*, and η is the QY, optical density, integrated emission intensity, and solvent refractive index, respectively. The subscript ‘c’ refers to the DP-CDs solution, and subscript ‘s’ refers to the quinine sulphate solution.

### Metal sensing of Fe^3+^ using DP-CDs in solution

To determine the sensing selectivity of the as-prepared DP-CDs towards metal ions, the FL intensity was measured in the presence of various aqueous metal chloride solutions with a concentration of 50 µM (Cr^2+^, As^2+^, Hg^2+^, Zn^2+^, Pd^2+^, Pd^2+^, Fe^3+^, Ba^2+^, Cu^2+^, Ag^+^, Mg^2+^, and Al^3+^) at room temperature^28^. The DP-CDs were diluted with distilled water to a concentration of 1 mg/ml. In the experiment, the DP-CDs solution was mixed with one of the above metal solutions in equal volumes, and the intensity was measured using a multilabel reader (Molecular Device Spectramax M3, Softmax Pro V5 5.4.1 software) at Ex_420 nm_ and Em_520 nm_. For the sensitivity assessment, the FL intensities of DP-CDs were calculated with different concentrations of Fe^3+^ to predict the limit detection limit^68^.

### Preparation of DP-CDs coated sensor-strip

For paper-based sensor strip preparation, the design of the microfluidic device was drawn on filter paper with a pencil (length diameter of reaction zone = 5 mm). A wax coating was made over the pencil marks with a wax marker. Subsequently, the wax-coated papers were placed on a hot plate at 120 °C for 2 min to fix the wax figure and pull in the hydrophobic boundaries. Subsequently, 20 µl of an aqueous DP-CDs dispersion was dropped within the boundaries on the filter paper and allowed to dry at room temperature^69^. The prepared sensor strips were now ready for the sensing application. Subsequently, 10 µl of a metal ion solution was placed on the sampling part and allowed to dry at room temperature. The FL intensity of the sampling area was observed using an ultraviolet inspection cabinet and captured with a Canon EOS 90D DSLR camera. Finally, the FL intensity of the captured images was measured using ImageJ software.

### Preparation and characterization of carbon dots-TiO_2_ nanocomposite (DCTN)

For DCTN preparation, 50 ml of a methanolic extract of *D. palmatus* and the DP-CDs solution (100 mg) were added to 50 ml of titanium isopropoxide (10 mM) and stirred for 30 min. The mixture was autoclaved at 120 °C for 30 min. A precipitate of the resultant-suspension was dried overnight at 180 °C. Finally, the resulting precipitate powder was calcined at 400 °C for 3 h. For material characterization, the structure of the prepared DCTN was examined by HR-TEM and FE-SEM. The 3D morphology and the size of the DCTN were observed by atomic force microscopy (AFM, Agilent Technology) in contact mode. Compositional characterization studies were performed with FT-IR spectroscopy and XPS. The absorbance characteristics of DCTN were analyzed by UV–Vis diffuse reflectance spectroscopy (UV-DRS) using a UV–Vis–NIR spectrophotometer (Shimadzu UV-3600).

### Photocatalytic bacterial deactivation of DCTN under sunlight

#### Assessing the bacterial growth of *V. harveyi* during DCTN photocatalysis

**Figure 8:**
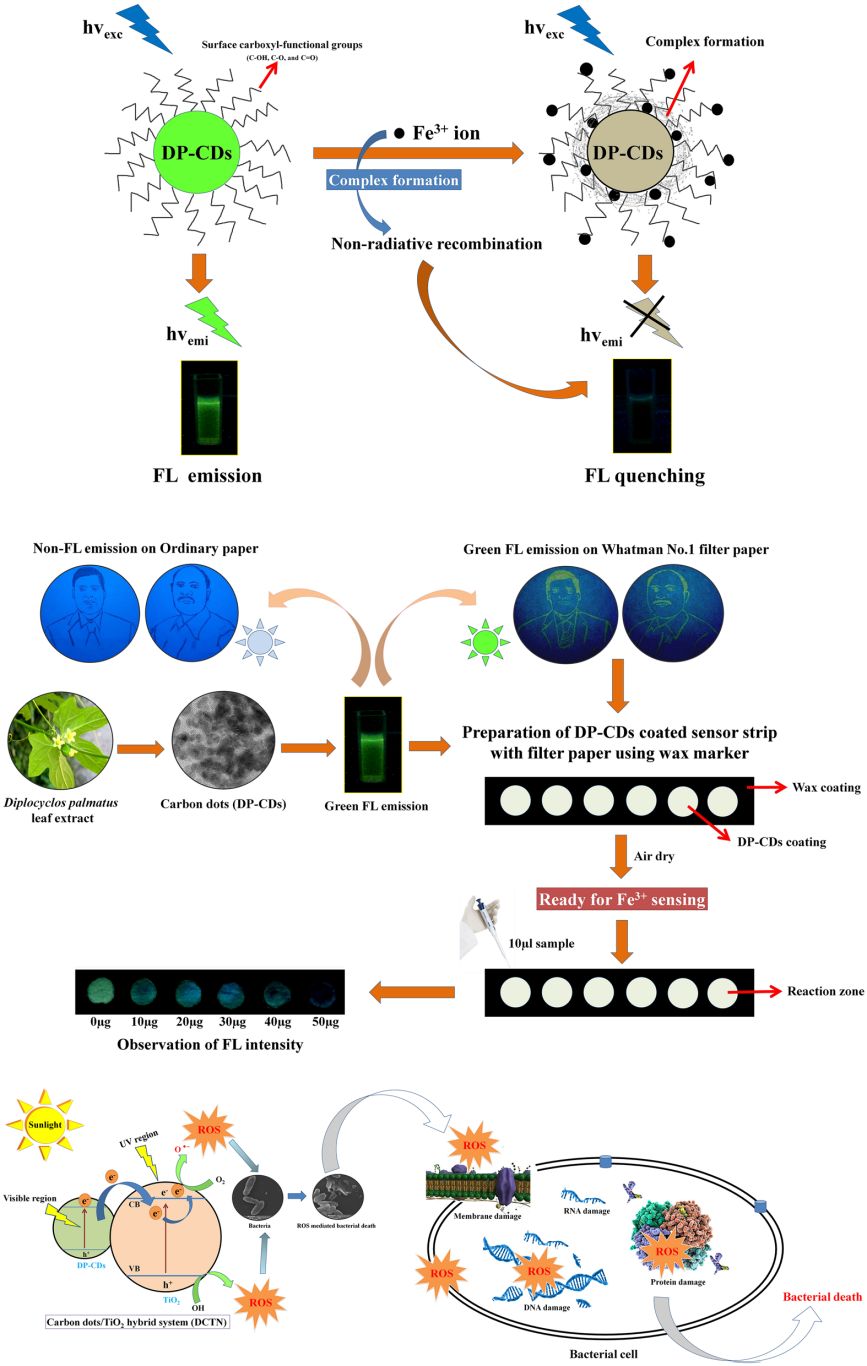
(**Scheme-1**) The possible mechanism of FL quenching of the DP-CDs upon binding with Fe^3+^ ions. (**Scheme-2**) The schematic representation of DP-CDs coated fluorometric sensor-strip preparation. (**Scheme-3**) Schematic diagram of the photocatalytic deactivation of *V. harveyi* using DCTN photocatalyst under sunlight irradiation.

The photocatalytic disinfection efficacy of DCTN was evaluated using *Vibrio harveyi* (MTCC 7771) as the model bacterium. The photocatalytic experiments were performed under direct sunlight irradiation between 11.00 am and 2.00 pm (light intensity was 983 ± 46 W m^−2^, measured using a photometer) from April to July. For this experiment, *V. harveyi* was cultured in 100 ml Luria Bertani (LB) medium overnight at 37 °C. The bacterial cells grown in the media were harvested using centrifugation and washed twice with PBS. The cell pellets were re-suspended in 100 ml of saline water (1% NaCl). Subsequently, 50 mg of DCTN was added to the *V. harveyi* suspension and placed in the dark for 10 min. The bacterial suspension with the DCTN photocatalyst was then exposed to direct sunlight. Every 60 min, 100 µl of the reaction samples were taken and spread on an LB agar plate. Each plate was incubated at 30 °C for 24 h, and the bacterial colonies were counted after the incubation period (Fig. 8).

#### FE-SEM analysis

For FE-SEM analysis, the bacterial samples were harvested using a centrifuge after 240 min sunlight irradiation. The bacteria were then fixed with a 2% glutaraldehyde solution for 4 h and washed twice with distilled water. The fixed samples were dehydrated with increasing concentrations of an ethanol solution (20, 40, 60, 80, and 100%) and dried overnight. Then, the prepared samples were observed in field emission scanning electron microscopy (FE-SEM) instrument (Zeiss ultra FE-SEM instrument).

#### Measurement of intracellular ROS in bacteria

The intracellular ROS generation in the bacterial cells was determined using the DCFDA method^70^. Briefly, 1 ml of treated and untreated culture was centrifuged at 5000 rpm for 15 min, and the supernatant was removed. Subsequently, 20 μg/ml of DCFDA was added and kept for 1 h at 37 °C. Finally, the fluorescence intensity was recorded at an excitation and emission wavelength of 485 nm and 535 nm, respectively, using a multilabel reader (Molecular Device Spectramax M3, Softmax Pro V5 5.4.1 software).

#### FT-IR analysis

In FT-IR analysis, the cell pellets of *V. harveyi* before and after photocatalysis were harvested and washed with 1% PBS. The KBr pellets were then prepared separately with these cell pellets. The FT-IR (NicoletTM iS5, Thermo Scientific, U.S.A) spectra were recorded in the range, 400–4000 cm^−1^, with a resolution of 4 cm^−1^ using OMNIC Software^71^.

#### ROS analysis in water under sunlight

An XTT assay and TA method were performed to confirm the generation of ROS in the photocatalytic reaction under sunlight irradiation^72^. In the 2,3-bis(2- methoxy-4-nitro-5-sulfophenyl)-2H-tetrazolium-5-carboxanilide (XTT) probe-based confirmation of O_2_·− generation, 100 µM XTT was used as an indicator. During the photoreaction, 1 ml of the experimental water was collected, and the intensity of the orange-colored XTT-formazan was measured at 470 nm using a multilabel reader (Molecular Device Spectramax M3, Softmax Pro V5 5.4.1 software). Terephthalic acid (10 μM, Sigma-Aldrich) was used as an indicator of OH· radical detection. In general, TA binds with OH· radicals to form the fluorescent product, 2-hydroxyterephthalic acid (hTA). Therefore, the fluorescence intensity of hTA was measured at Ex _310 nm_ and Em_420 nm_ using a multilabel reader.

### Assessing the anti-infection efficacy of DCTN against *V. harveyi-*caused AHPND

#### Biofilm assay

In biofilm assay, 10 µl of an overnight culture of *V. harveyi* was added to 1 ml of sterile LB broth in 24-well MTP containing different concentrations of DCTN (10–50 µg/ml). After 24 h incubation, the planktonic cells were discarded, and adhering biofilm cells were mildly rinsed with distilled water. Then, the biofilm cells were stained with 0.2% crystal violet (Himedia, India) and suspended in 1 ml of 20% glacial acetic acid. Finally, the intensity was measured at OD_570_ nm using UV–Vis spectroscopy and calculated the inhibition percentage^73^.

#### In-situ visualization of biofilms

For light microscopic analysis, the biofilm assay was carried out with glass slides (1 × 1 cm). Then, the glass slides were rinsed twice with distilled water and stained with 0.2% crystal violet. After that, the glass slides were observed under a light microscope with an attached digital camera (Nikon Eclipse Ti 100) at 400 × magnification^74^. For confocal laser scanning microscopy (CLSM) image analysis, the biofilms were stained with 0.1% acridine orange and observed under CLSM (Zeiss LSM 710, Carl Zeiss, Germany) at the magnification of 200 ×.

#### Hemolysin assay

In hemolysin assay, 10 µL of *V. harveyi* culture was inoculated into 1 ml of LB broth containing the various concentrations of DCTN (10–50 µg/ml) and incubated at 28 °C for 24 h. Then, the cell-free culture supernatants (CFCS) were collected using centrifugation. After, 100 µl of CFCS was added to 900 µl of 2% sheep erythrocytes in phosphate buffer saline (PBS). The mixture was placed in ice for 20 min and centrifuged to collect supernatants. Finally, the absorbance of hemoglobin was measured at 530 nm using a UV–Vis spectroscopy^23,24^.

#### Protease assay

In protease assay, 10 µl of *V. harveyi* culture was inoculated into 1 ml of LB broth containing the different concentrations of DCTN (10–50 µg/ml) and incubated at 28 °C for 24 h. After incubation time, the 75 µl of CFCS was added to 125 µl of 2% azocasein in 0.25 M Tris (pH 8.0) and incubated for 30 min at 37 °C. Subsequently, the reaction was stopped using 10% trichloroacetic acid and the supernatant was collected through centrifugation. Finally, the absorbance of the supernatant was measured at 440 nm using a UV–Vis spectroscopy^24^**.**

#### Toxicity assessment using *P. vannamei* survival assay

Healthy Whiteleg shrimps (*P. vannamei*) (body length: 4.5 ± 0.26 cm) were purchased from Allwin hatchery, Pondicherry, India and acclimatized for a week. Then, ten numbers of shrimp were separately transferred to 20 l plastic bucket containing different concentrations of DCTN, P25-TiO_2_, and DP-TiO_2_ in 5 l of sterile seawater (30 ppt) at 27 °C. Without any material supplement considered as normal control. The mortality was counted and the survival percentage was calculated after 96 h of the experiment for assessing their toxicity level. Experiments were performed triplicates and no feeding was provided during the experiments^61^.

#### *V. harveyi* challenge study

For in vivo challenge test, shrimps were infected with 1 × 10^8^ CFU/ml for 4 h. Then, the infected shrimps were transferred into a new 20L plastic bucket containing 12.5 µg/ml of DCTN in 5 l sterile seawater, named as treated group. Without DCTN treatment is considered an infected group. The mortality of experimental animals was counted and the survival percentage was calculated after 96 h. The experiments were performed triplicates and no feeding was provided during the experiments^61^. To assess the reduction of *V. harveyi* colonization, the shrimp hepatopancreas HP was collected and homogenized with sterile PBS. Then, the homogenized samples were allowed to spread plating and the colonies were counted after incubation^23^.

### Histopathology analysis

The experimented shrimps were collected and immediately fixed in 10% (v/v) phosphate-buffered formalin containing 4 g/l of NaH_2_PO_4_, 6.5 g/l Na_2_HPO_4_ in 100 ml of 40% formalin in 900 ml of distilled water. Then, the shrimps were preserved in 70% ethanol till processing. Then, the formalin-fixed animals were allowed to sectioning using a microtome and tissue embedding. Finally, the cut sections were subjected to hematoxylin and eosin (H&E) staining and they were observed under a light microscope with an attached digital camera (Nikon Eclipse Ti 100)^65^.

## Supplementary information

Supplementary Figures.
