# Supplementary material for: Metal sensing-carbon dots loaded TiO2-nanocomposite for photocatalytic bacterial deactivation and application in aquaculture
Source: Sci Rep. 2020 Jul 30;10:12883. doi: 10.1038/s41598-020-69888-x (PMC7393085; doi:10.1038/s41598-020-69888-x)

Supplementary information

Metal sensing-carbon dots loaded TiO_2-nanocomposite for photocatalytic bacterial deactivation and application in aquaculture

Rajaiah Alexpandi[1], Chandu V.V. Muralee Gopi[2], Ravindran Durgadevi[1], Hee-Je Kim[2], Shunmugiah Karutha Pandian[1], and Arumugam Veera Ravi[1]*

[1] Lab in Microbiology and Marine Biotechnology, Department of Biotechnology, School of Biological Sciences, Alagappa University, Karaikudi-630 003, India.

[2] Lab in Laser and Sensor Application, School of Electrical and Computer Engineering, Pusan National University, Busandaehak-ro 63 beon-gil, Geumjeong-gu, Busan 46241, South Korea.

***Corresponding Author:**

Dr. A. Veera Ravi,

Professor,

Lab in Microbiology and Marine Biotechnology,

Department of Biotechnology,

School of Biological Sciences,

Alagappa University,

Karaikudi- 630 003, India.

E-mail: aveeraravi@rediffmail.com

Tel: +91 4565 223323; Fax: +91 4565 225202

Supplementary Figures

Fig. S1 The FL stability of as-prepared DP-CDs under different temperatures.

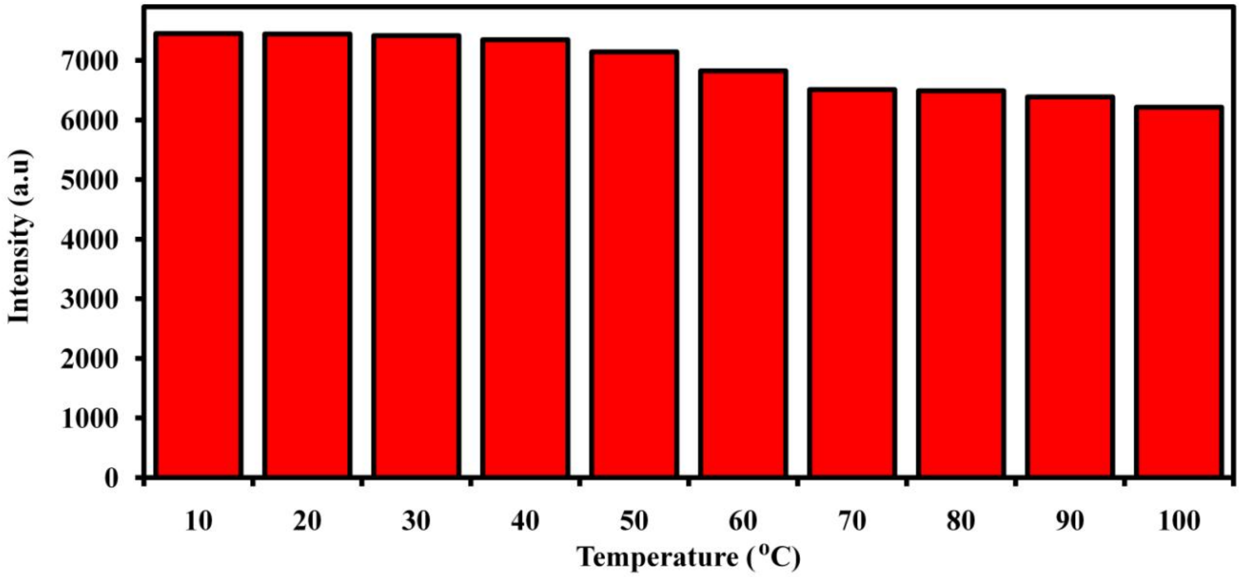

Fig. S2 The FL stability of as-prepared DP-CDs under various solutions.

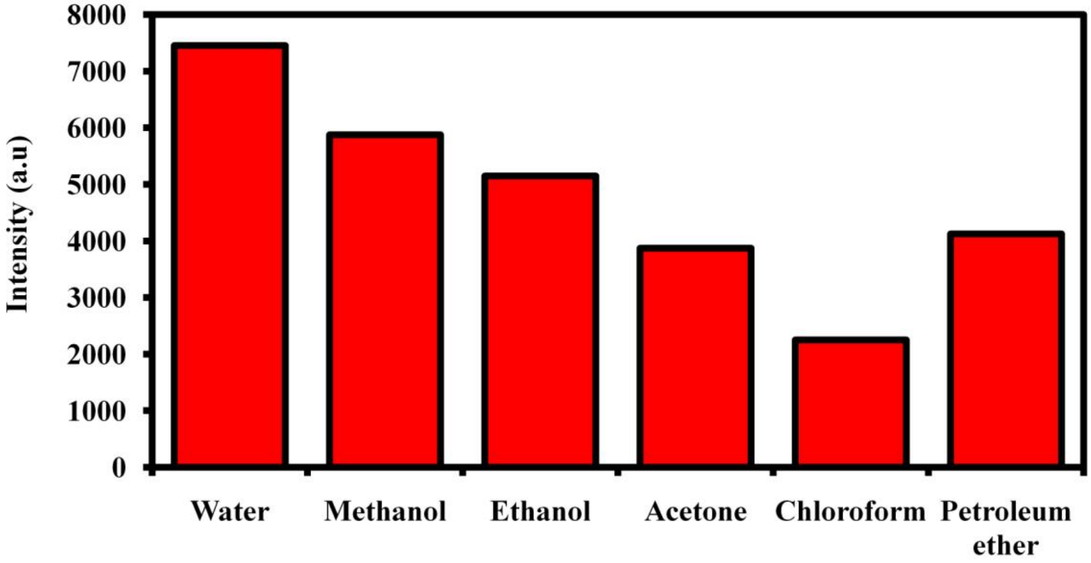

Fig. S3 (a) 2D image of surface morphology of DCTN. (b) 3D image of the DCTN spheres. (c) Size measurement of DCTN using AFM analysis.

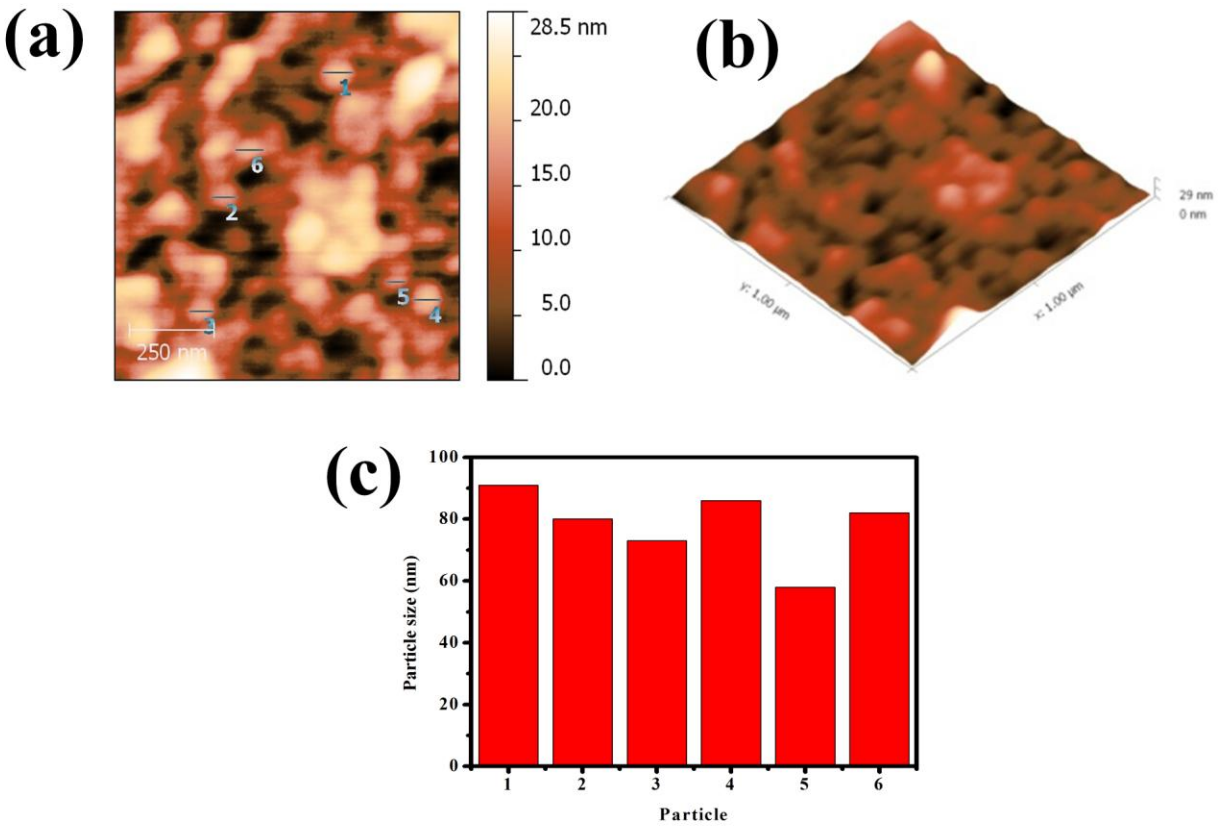

Fig. S4 XRD analysis of (a) DP-TiO_2 (b) DCTN (c) P25 TiO_2.

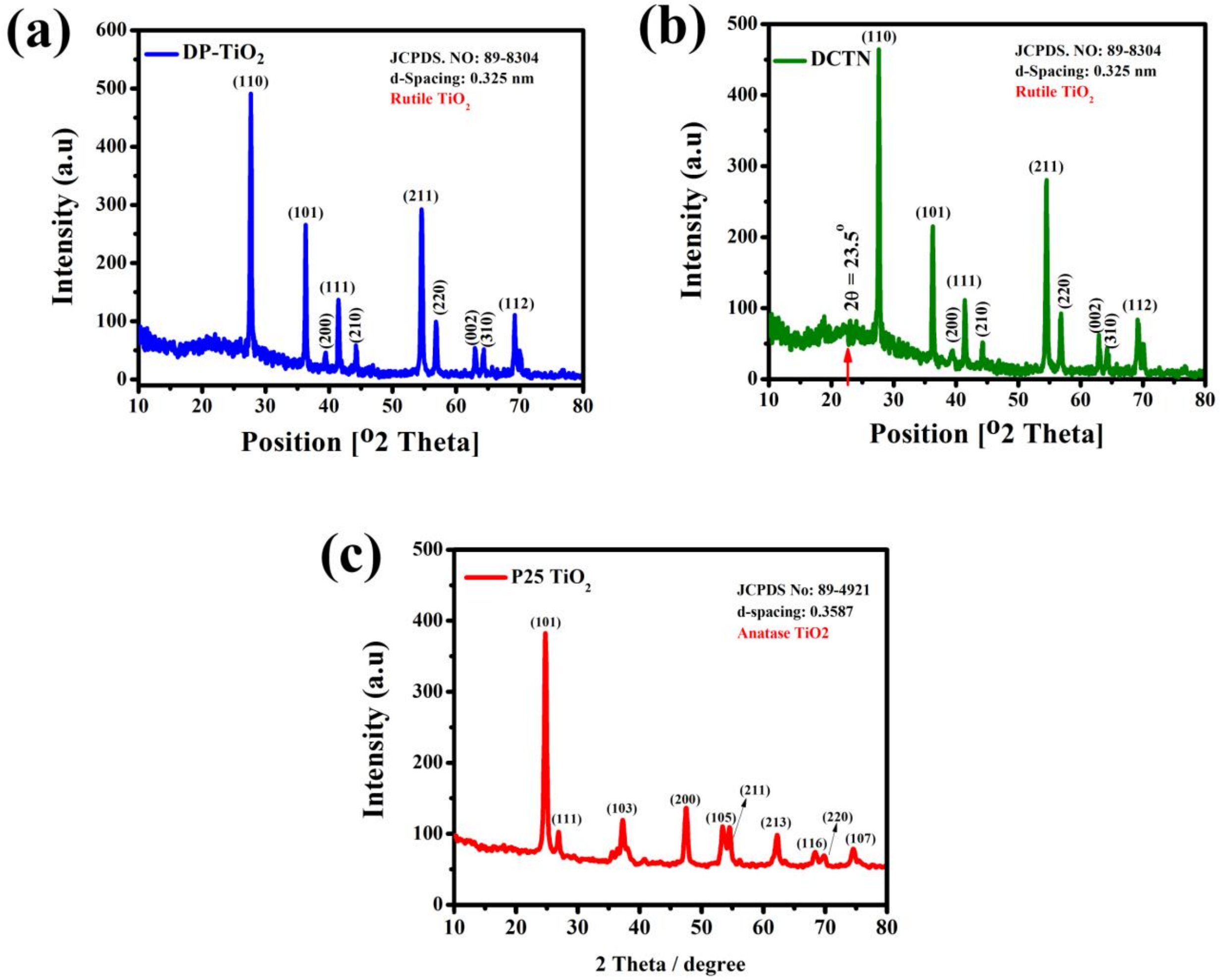

Fig. S5 Estimated Bandgap of (a) DP-TiO_2 and (b) DCTN.

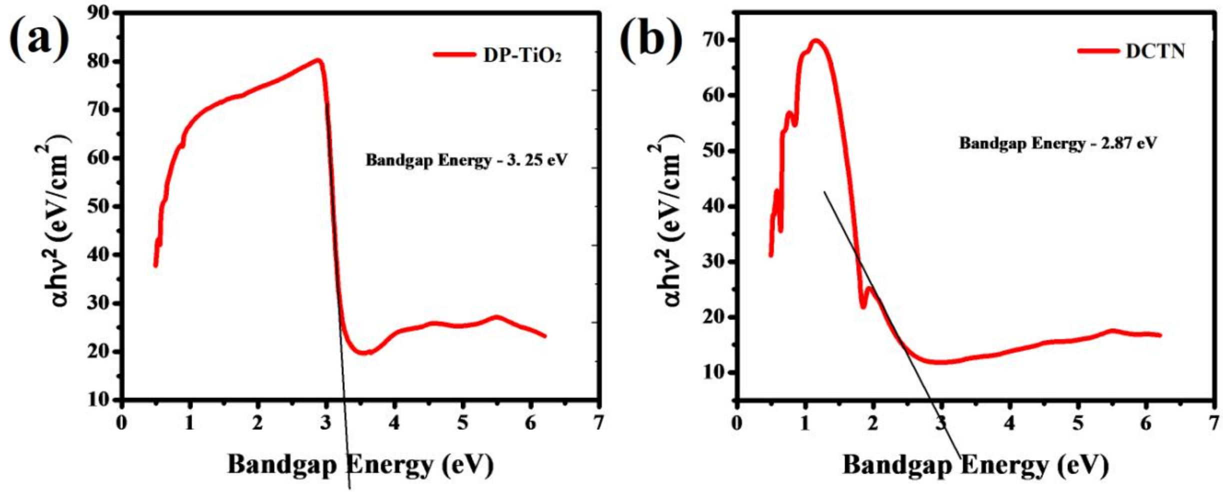

Fig. S6 Photocatalytic bacterial deactivation of *V. harveyi* under sunlight irradiation.

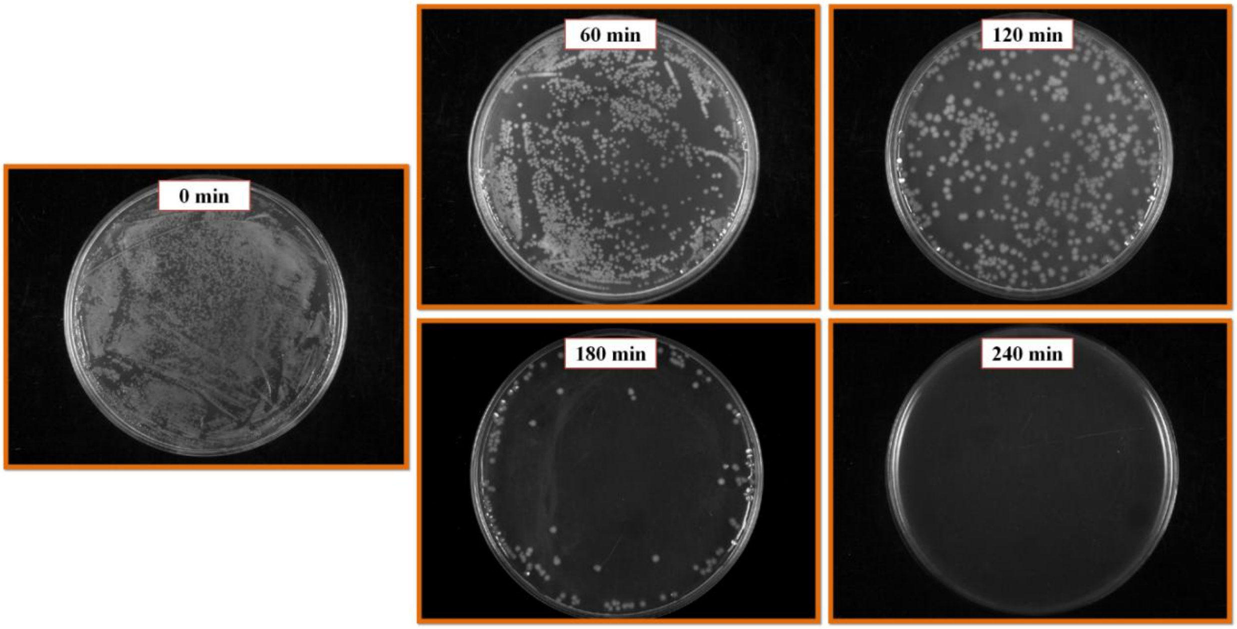

Fig. S7 Photocatalytic bacterial deactivation of natural seawater using DCTN under sunlight irradiation.

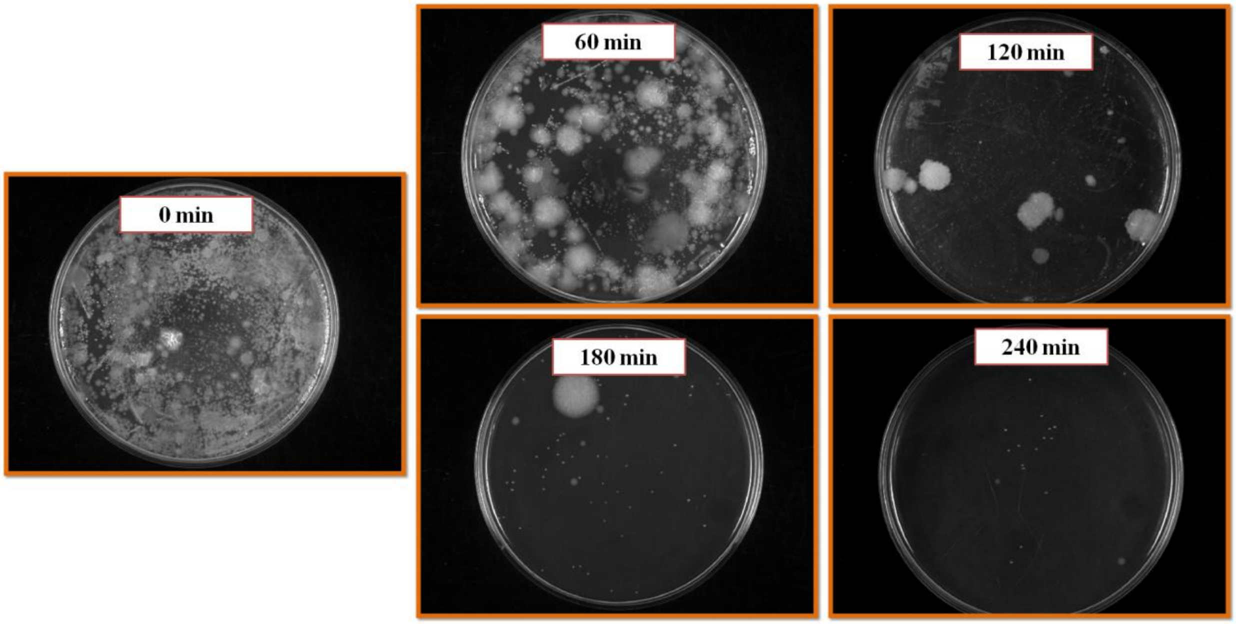

Fig. S8 Photocatalytic bacterial deactivation of tap water under sunlight irradiation.

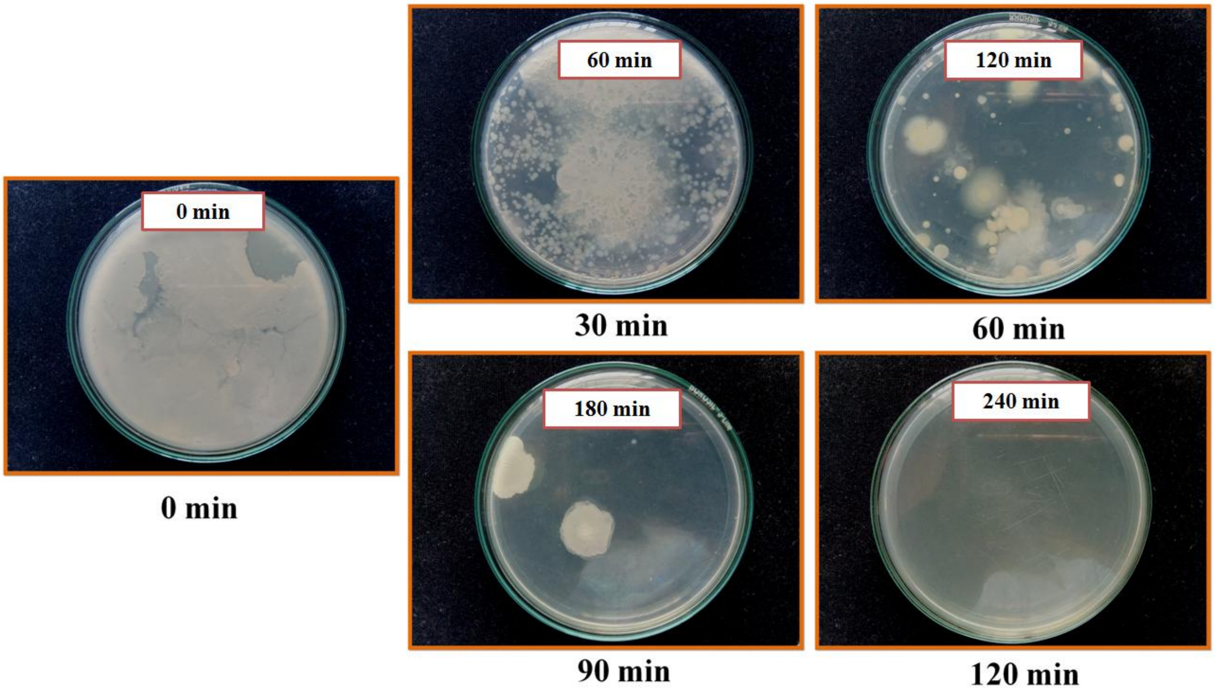

Fig. S9 Identification of O_2•− radicals formation in water during DCTN photocatalysis by XTT method.

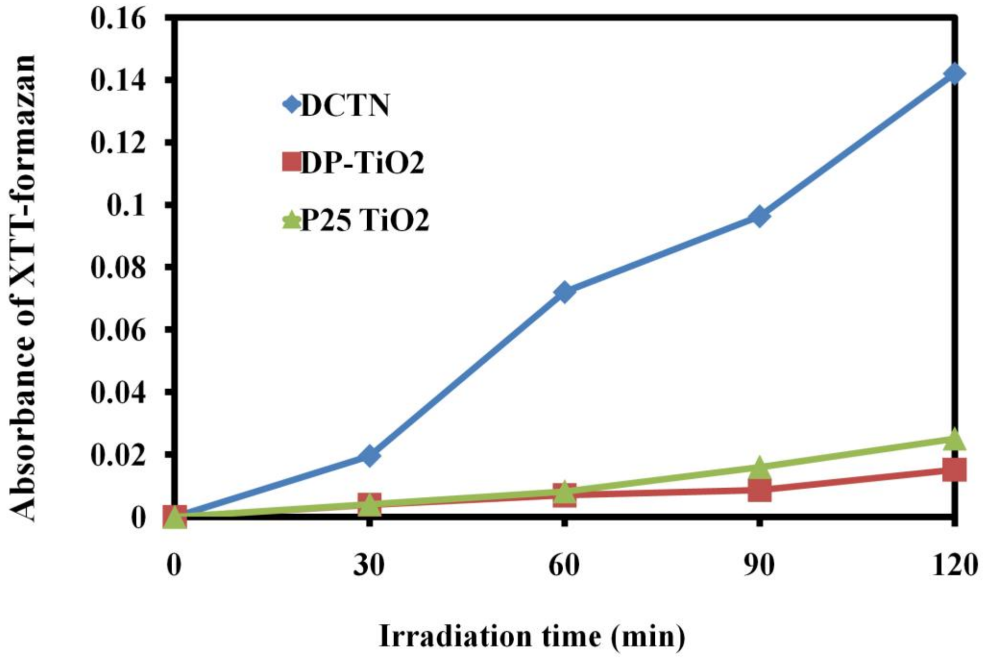

Fig. S10 Identification of OH• radicals generation in water during DCTN photocatalysis by TA method.

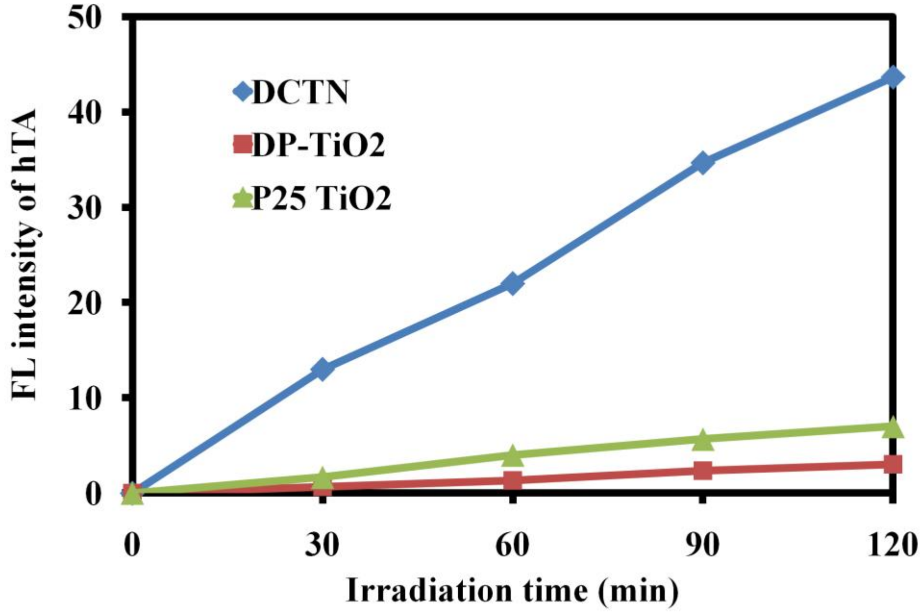

Fig. S11 Inhibition percentage of hemolysin and protease in *V. harveyi* upon DCTN treatment.

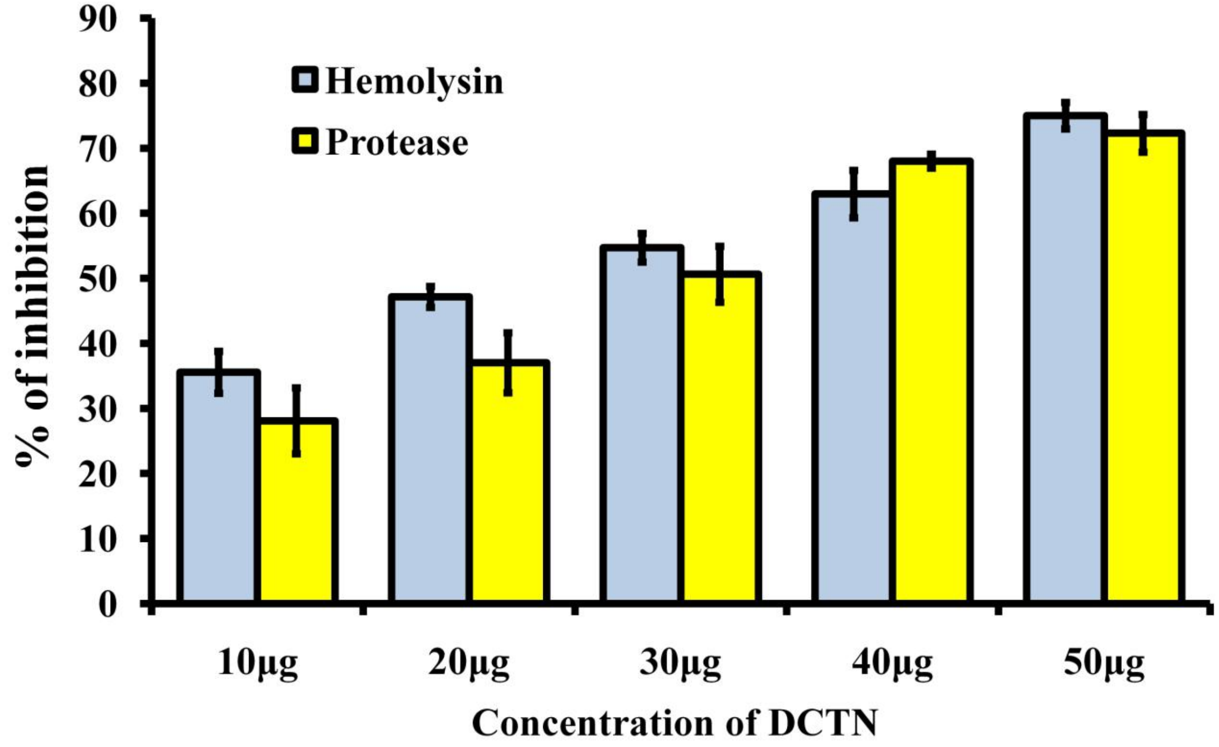

Fig. S12 The *in vivo* toxicity assay reveals the survival percentage of shrimps in the presence of DCTN, P25-TiO_2, and DP-TiO_2 after 96 h.

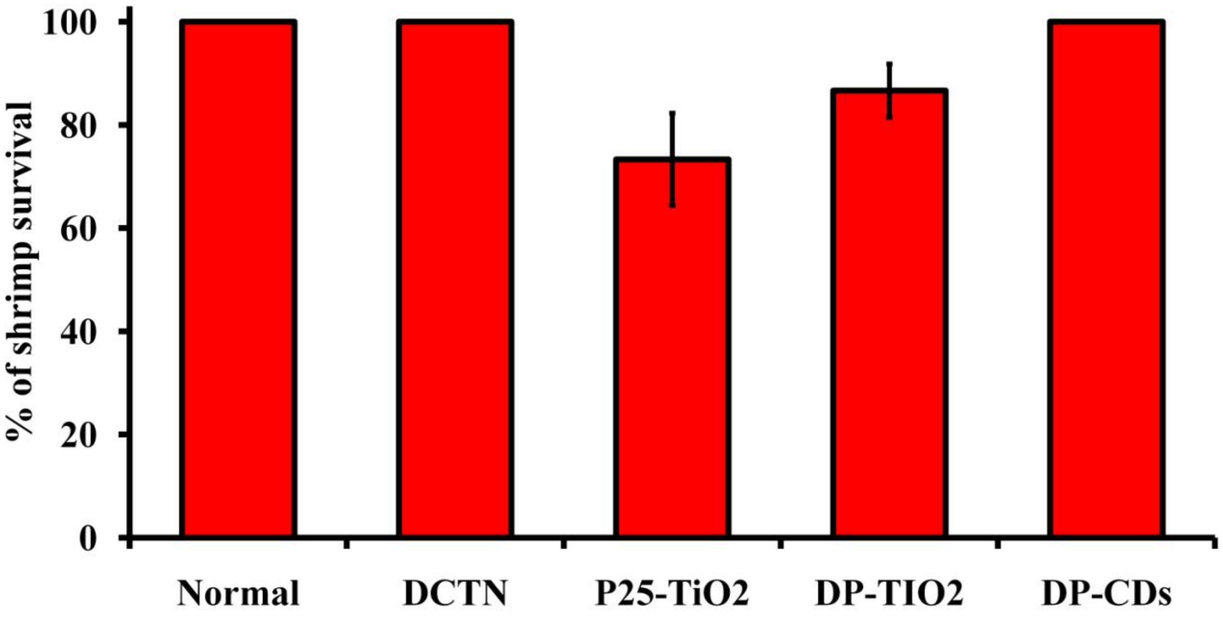

Supplement: Supplementary file 1 — Supplementary Figures. [file 41598_2020_69888_MOESM1_ESM.pdf]
